# Distinct mode of action of a highly stable, engineered phage lysin killing Gram-negative bacteria

**DOI:** 10.1128/spectrum.01813-23

**Published:** 2023-11-16

**Authors:** Hans Gerstmans, Lisa Duyvejonck, Roberto Vázquez, Ines Staes, Jimmy Borloo, Karim Abdelkader, Jeroen Leroy, Emma Cremelie, Diana Gutiérrez, Héctor Tamés-Caunedo, Patricia Ruas-Madiedo, Ana Rodríguez, Abram Aertsen, Jeroen Lammertyn, Rob Lavigne, Yves Briers

**Affiliations:** 1 Department of Biotechnology, Ghent University, Ghent, Belgium; 2 Department of Biosystems, KU Leuven, Leuven, Belgium; 3 Department of Microbial and Molecular Systems, Leuven, Belgium; 4 VIB Discovery Sciences, VIB, Ghent, Belgium; 5 Department of Microbiology and Immunology, Beni-Suef University, Beni-Suef, Egypt; 6 Dairy Research Institute of Asturias, Spanish National Research Council (IPLA-CSIC), Villaviciosa, Asturias, Spain; The University of North Carolina at Chapel Hill, Chapel Hill, North Carolina, USA

**Keywords:** lysin, mode of action, *Acinetobacter baumannii*, serum, osmotic lysis, thermoresistance

## Abstract

Therapeutic options to treat bacterial infections caused by Gram-negative pathogens are limited due to the spread of multidrug resistance. Protein engineering of phage-derived lysins can play a key role in the search for new antimicrobial compounds targeting Gram-negative pathogens. A previous high-throughput screen of a combinatorial lysin library identified lysin 1D10 active against *Acinetobacter baumannii* under elevated human serum concentrations. The engineered lysin consists of four modules: cecropin A, a linker, cell wall-binding domain, and an enzymatic active domain. Using time-lapse microscopy, we show that 1D10 has a distinct antibacterial mode of action resulting in local cell wall bulging at the septum instead of cell-wide lysis, as observed for previously reported engineered lysins that target Gram-negative bacteria. Our results indicate that the activity of 1D10 relies on the antibacterial activity of both cecropin A (CecA) and the enzymatically active domain. Based on a truncation analysis, the role of each of the four modules of 1D10 was dissected. We further compared the antibacterial spectrum, thermostability, and cytotoxicity of cecropin A alone and lysin 1D10. Both lysin 1D10 and CecA are most active against *A. baumannii* and are not cytotoxic toward human keratinocytes. Lysin 1D10 unfolds at 57°C and has a remarkable refolding capacity, as it regains its activity even after exposure to 90°C and sterilization conditions, whereas CecA is inactivated at 70°C. Overall, the present study shows that an improved understanding of the killing mechanism and the protein properties will further support lysin engineering designs in the future.

**IMPORTANCE:**

Engineered lysins are considered as highly promising alternatives for antibiotics. Our previous screening study using VersaTile technology identified 1D10 as a possible lead compound with activity against *Acinetobacter baumannii* strains under elevated human serum concentrations. In this manuscript, we reveal an unexpected mode of action and exceptional thermoresistance for lysin 1D10. Our findings shed new light on the development of engineered lysins, providing valuable insights for future research in this field.

## INTRODUCTION

Antimicrobial resistance is a global concern due to its large impact on community health and healthcare systems, mainly as a consequence of the reduced number of treatment options (1 – 3). Lysins are peptidoglycan-degrading enzymes produced by (bacterio)phages to break the cell wall barrier at the initiation and the end of the lytic cycle. Exogenous application of purified lysins kills Gram-positive cells. In view of their clinical progress in recent years, they are considered as one of the most important alternative classes of antibacterials (4, 5). Lysins, however, are generally too large to pass the outer membrane (OM) barrier of Gram-negative bacteria. Protein engineering by fusing outer membrane permeabilizing peptides (6), pyocin domains (i.e., lysocins) (7), or receptor-binding proteins of phages (i.e., innolysins) (8) to lysins has been successfully demonstrated to facilitate passage through the OM and to kill Gram-negative bacteria. To date, data concerning the activity of these engineered lysins in complex biological media such as human serum, blood, urine, or sputum remain scarce (9).

**Fig 1:**
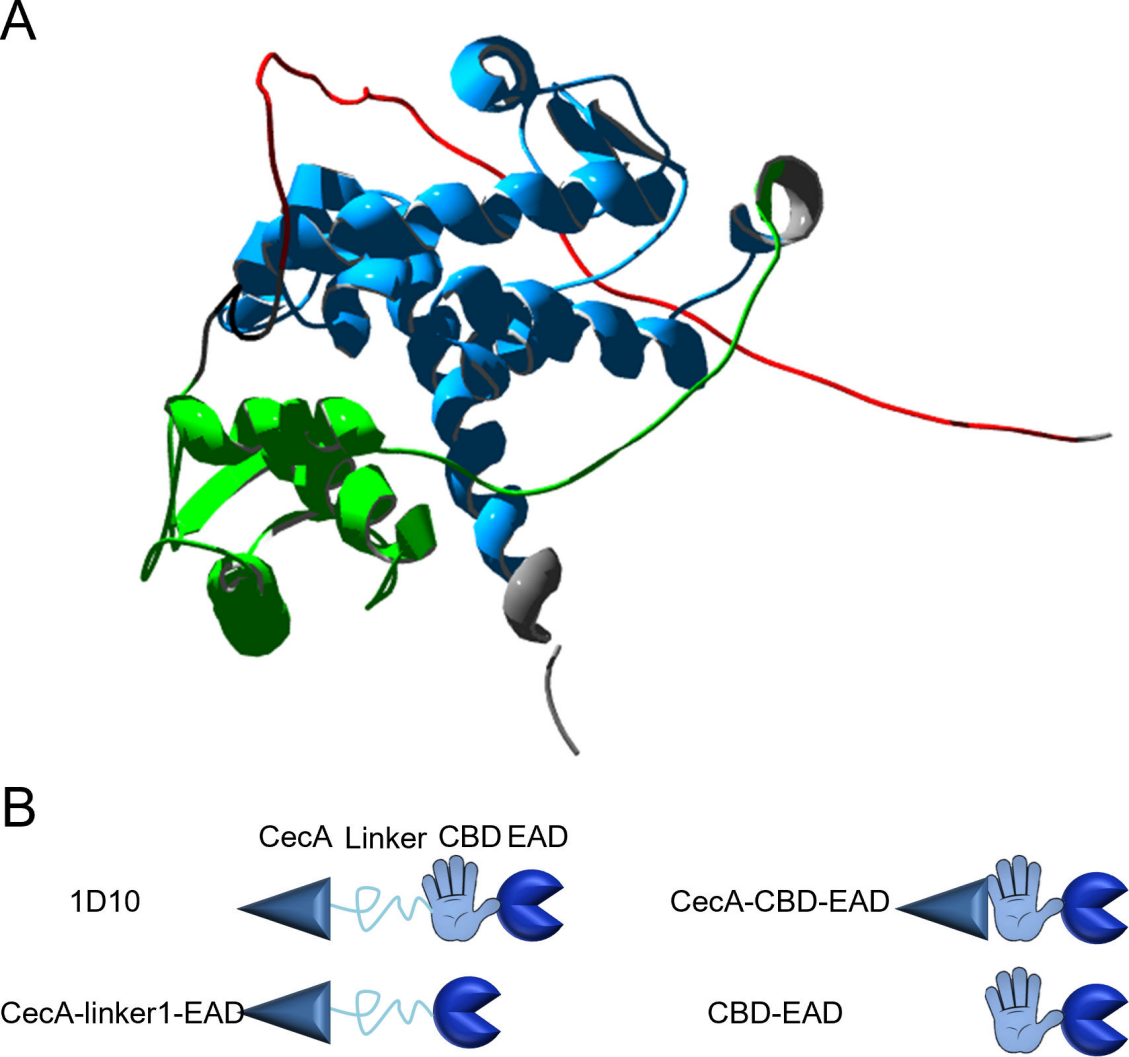
AlphaFold model of 1D10 and graphical representation of derivatives to elucidate the function of the linker and CBD. (**A**) Model of 1D10 made by AlphaFold, depicting the different domains to their actual scale (red = CecA, black = linker, green = CBD, blue = EAD, gray = VersaTile scars). (**B**) On the top left, the complete composition of lysin 1D10 is shown. In all other constructs, one or more building blocks are absent. The size of the different building blocks in B is not drawn to scale (CecA: 36 aa, linker1: 6 aa, CBD: 82 aa, EAD: 137 aa).

We recently developed the VersaTile technique, which enabled us to construct and screen a combinatorial library of modular, engineered lysins (*n* = 9,576), each variant comprising an outer membrane permeabilizing (OMP) peptide, a linker, a cell wall binding domain (CBD), and an enzymatically active domain (EAD) in this respective order (10). *A. baumannii* was selected as a target because this pathogen causes several healthcare-associated infections for which only a limited number of treatment options are available. Iterative screening for potent lysin activity against *A. baumannii* strains under human serum conditions was performed. In the first two rounds, we enriched the library for variants with high activity against *A. baumannii*. The cecropin A (CecA) peptide was most prevalent among those hits. We subsequently analyzed the best 24 variants in 50% human serum. Five of them showed activity in human serum, all of them containing the CecA peptide. We eventually identified variant 1D10 as the most outstanding variant. This lysin 1D10 consists of (i) the OMP peptide CecA produced by the yellow fever mosquito, *Aedes aegypti* (11); (ii) a linker of three Ala-Gly-repeats with predicted neutral, short, and flexible properties; (iii) the modified CBD of the endolysin of bacteriophage ϕKZ (12) (including the natural linker at the N-terminal side); and (iv) the EAD of gp16 located at the C-terminus of the virion-associated lysin of phage BcepC6B (10) (Fig. 1; Fig. S1). In this study, we describe a distinct mode of action for lysin 1D10 compared to previously reported engineered lysins targeting Gram-negative bacteria, as well as an unusually high thermoresistance.

## RESULTS

### Lysin 1D10 has a unique mode of action

**Fig 2:**
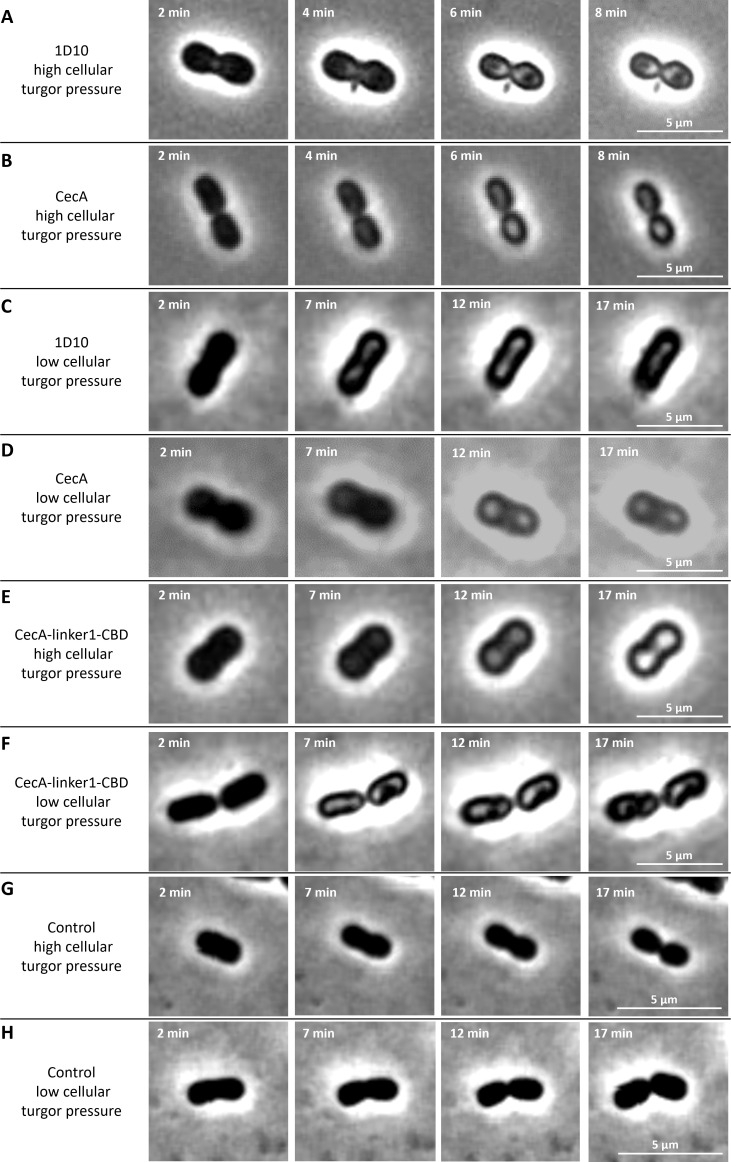
Time-lapse microscopy of the antibacterial mode of action of lysin 1D10, CecA, and CecA-linker1-CBD against *A. baumannii* RUH134 with high and low turgor pressure. Cells with a high turgor pressure using agar pads prepared with dilute buffer (**A, B, and E**) or a low turgor pressure using agar pads prepared with human serum were evaluated (**C, D, and F**). The effect of lysin 1D10 (**A–C**) was compared visually to the effects of CecA (**B–D**) and CecA-linker1-CBD (**E–F**). In all cases, a gradual change in phase contrast was observed, but when lysin 1D10 was added against cells with a high turgor pressure, apparent, local releases of cytoplasmic content by cell wall bulging were observed at the septum. Untreated controls showed active growth and replication under conditions of both low (**G**) and high intracellular turgor pressure (**H**). Equimolar amounts of each compound (2 µM) were used. Representative cells are shown for each condition. The interval between each panel is indicated in the left, top corner. Each scale bar measures 5 µm. Lysin 1D10 consists of (i) the OMP CecA; (ii) a neutral, short, and flexible linker; (iii) the modified CBD; and (iv) an EAD. All other constructs lack one or more parts of 1D10.

Previously reported engineered lysins targeting Gram-negative bacteria kill the cells by complete osmotic lysis (6, 12, 13). Based on a series of time-lapse microscopy experiments with lysin 1D10, we observed no full cell lysis but a distinct killing mechanism (Movie S1; Fig. 2A). Exposure to lysin 1D10 induces cell wall bulging at the septum, apparently releasing cytoplasmic content. This is followed by a changing phase contrast of the cells, indicating a substantial spatial redistribution of the cellular content. All these effects take place within a short timespan (<10 min). In some cells (≤10%, Fig. S2), cell wall bulging starts at the poles instead of the septum (Movie S2). When repeating the assay with an equimolar amount of the OMP peptide, CecA, alone (Movie S3; Fig. 2B), a similar change in phase contrast was observed, yet without cell wall bulging. Since cecropins are known to form pores or carpet-like structures that destabilize and depolarize the membrane (14, 15), these microscopic observations may be associated with membrane depolarization.

Notably, these time-lapse series were monitored on agar pads that were prepared with dilute buffer, resulting in a high intracellular turgor pressure. When both experiments were repeated on serum agar pads that present more isotonic conditions for the cells and thus result in a lowered intracellular turgor pressure (Movie S4 and S5; Fig. 2C and D for lysin 1D10 and CecA, respectively), only changes in phase contrast were observed for both lysin 1D10 and CecA. This suggests that cell wall bulging results from the concerted action of peptidoglycan degradation by the EAD moiety and a sufficiently high turgor pressure. When analyzing a truncated variant CecA-linker1-CBD, lacking the enzymatic activity of the EAD, cell wall bulging could not be observed under both high (Movie S6; Fig. 2E) and low turgor pressures (Movie S7; Fig. 2F). While these observations point to the role of the EAD in cell wall bulging, we cannot fully exclude that the lack of cell wall bulging is due to inappropriate folding of the truncated variant CecA-linker1-CBD.

### A low specific activity of 1D10 and its relationship to bacterial lytic transglycosylases involved in cell wall remodeling

An activity assay performed on fluorescently labeled cell wall substrate (EnzChek Lysozyme Assay Kit) confirmed the ability of 1D10 to degrade peptidoglycan (Fig. S3). However, a standard turbidity reduction assay performed with outer membrane-permeabilized *A. baumannii* RUH134 cells did not result in apparent lysis (Fig. S4) (16, 17). Taken together with the time-lapse microscopy, these results suggest that the peptidoglycan-degradation activity of the EAD in 1D10 is localized or restricted. A further confirmation of the latter is supported by the low specific activity displayed by 1D10 (3.10 ± 1.85 × 10^−3^ U µmol^−1^ min^−1^) compared to that of hen egg white lysozyme (1.27 ± 0.70 × 10^−2^ U µmol^−1^ min^−1^), both calculated from the data shown in Fig. S3. While the EAD in 1D10 is predicted to belong to a family of known glycosidases [Pfam *SLT* (PF01464) family, e-value = 1.3 × 10^−11^], the phylogenetic tree in Fig. S5 shows that it is associated closer to bacterial lytic transglycosylases (bLTs) than to g-type lysozymes, both members of *SLT* families. Besides, in general, the virion-associated lysins from the *SLT* family cluster together with the EAD from 1D10 and bLTs, while *SLT* endolysins are primarily located at the g-type lysozymes clade. This may imply a functional convergence between *SLT* virion-associated lysins (including the EAD of 1D10) and bLTs in the sense that their lytic transglycosylase activity is not meant to critically damage the bacterial cell wall, but rather to transiently remodel it, providing anhydro bonds that can act as acceptors of new material for repair or further remodeling (18, 19). By contrast, endolysins of the same family have evolutionarily diverged, perhaps to acquire an increased catalytic efficacy. This provides a theoretical framework to explain the low specific activity of 1D10.

### Peptidoglycan degradation and turgor pressure act in concert

**Fig 3:**
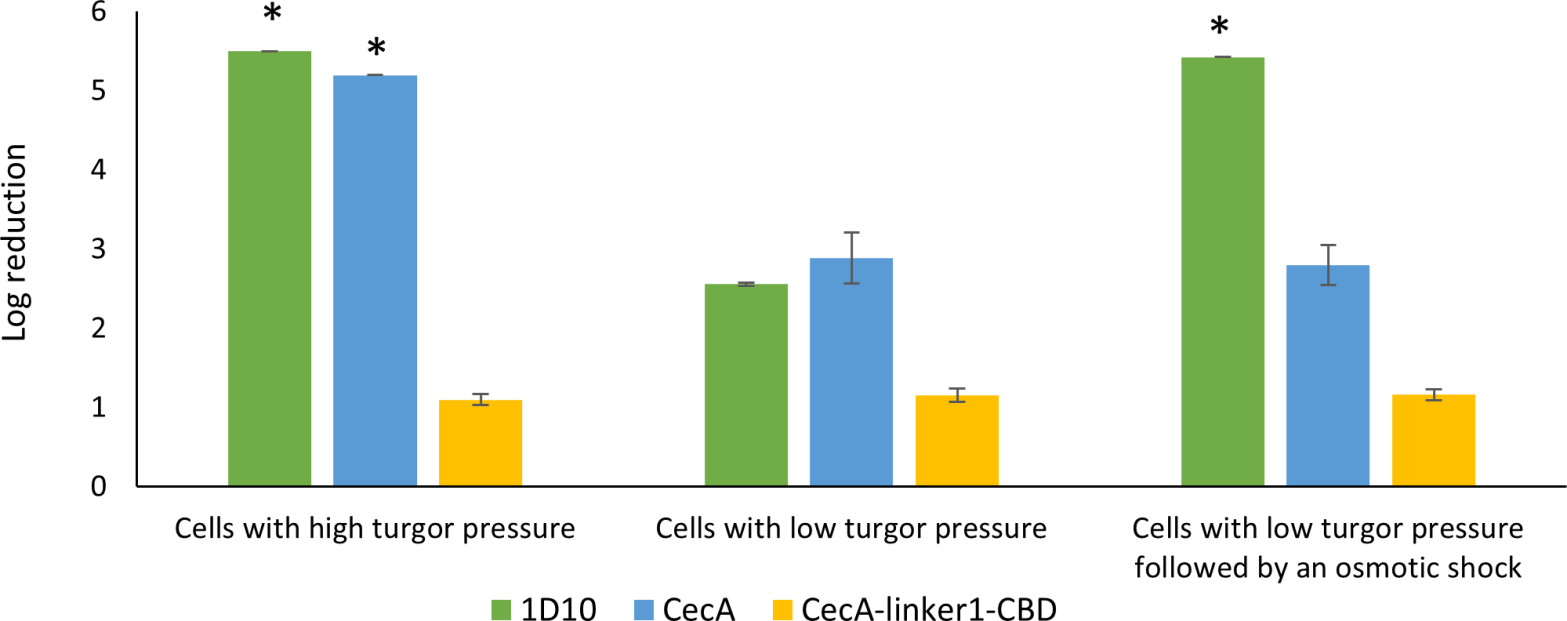
Antibacterial activity of equimolar concentrations of lysin 1D10, CecA and CecA-linker1-CBD against cells with high turgor pressure (dilute buffer) and cells with low turgor pressure (serum) with and without osmotic shock. Lysin 1D10 (green bars) and CecA (blue bars) cause more than 5 log reduction of cells with high turgor pressure (detection limit of the assay). Their antibacterial activity is reduced to 2.56 and 2.89 log units against cells with lowered turgor pressure, respectively. Lysin 1D10 reaches again the detection limit when the bacterial cells are exposed to an osmotic shock after treatment in contrast to CecA. The activity of CecA-linker1-CBD (yellow bars) was low and comparable under all conditions (1.1 log). Log reductions are calculated for each compound compared to the respective untreated control. Asterisk (*) indicates that the detection limit of the assay was reached.

Having proven the presence of an enzymatic, peptidoglycan degradation activity in 1D10, the absence of local cell wall bulging under low turgor pressure cells exposed to lysin 1D10 may be due to either a lack of enzymatic activity of the EAD under serum conditions or a lack of a sufficiently high turgor pressure. Therefore, quantitative killing assays were performed under three different conditions: (i) cells suspended in dilute buffer (high intracellular turgor pressure) followed by exposure to equimolar amounts of lysin 1D10, CecA, and CecA-linker1-CBD and dilution with the same dilute buffer before plating; (ii) cells resuspended in serum (low intracellular turgor pressure), followed by exposure to equimolar amounts of lysin 1D10, CecA, and CecA-linker1-CBD and dilution with the same serum before plating; and (iii) cells resuspended in serum, followed by exposure to equimolar amounts of lysin 1D10, CecA, and CecA-linker1-CBD and dilution with dilute buffer before plating. The latter condition creates an osmotic shock for the cells, lysing cells that are damaged sublethally by peptidoglycan degradation (Fig. 3). Lysin 1D10 and CecA show a similar killing effect with full eradication (≥5.5 and ≥5.2 log reduction, respectively) within 1 hour when cells are placed under high turgor pressure. However, the activity of CecA-linker1-CBD shows significantly lower activity with a 1.1 ± 0.1 log reduction of the same cells (*P* < 0.005) under the same conditions. The activity of both lysin 1D10 (2.6 ± 0.1) (*P* < 0.005) and CecA (2.9 ± 0.3) (*P* < 0.005) is significantly decreased when the turgor pressure is reduced. By contrast, the activity of CecA-linker1-CBD remains unaffected. When bacterial cells treated under low turgor pressure are exposed to an osmotic shock after treatment, the antibacterial activity of 1D10 again reaches maximal log reduction (≥5.4). By contrast, the antibacterial activity of CecA and CecA-linker1-CBD remains unaffected (2.8 ± 0.3 and 1.1 ± 0.1 log reduction (*P* < 0.005), respectively) compared to cells without osmotic shock. Since an osmotic shock kills cells when the integrity of the peptidoglycan sacculus is compromised, these observations indicate that at least partial peptidoglycan degradation has taken place under serum conditions.

### The outer membrane is effectively permeabilized

**Fig 4:**
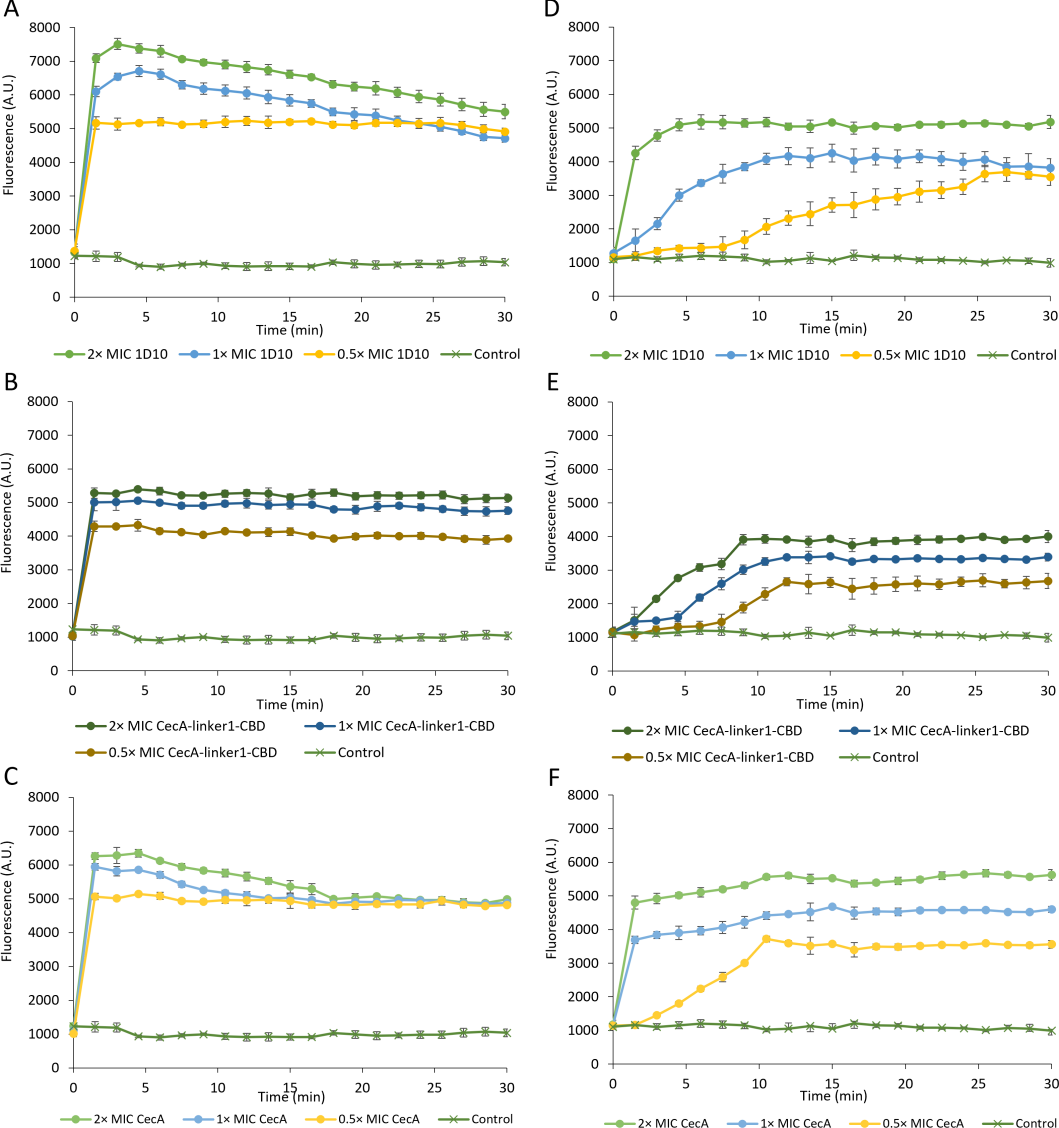
Measurement of OM permeabilization upon exposure to lysin 1D10 and its derivatives. (A–C) Cells suspended in a dilute buffer (high intracellular turgor pressure). (D–F) Cells resuspended in the same buffer supplemented with 150 mM NaCl (low intracellular turgor pressure). Lysin 1D10 (**A and D**), CecA-linker-CBD (**B and E**), and CecA (**C and F**) are tested with equimolar concentrations, referring to the minimum inhibitory concentration (MIC) of lysin 1D10 (2× MIC, filled light green circles; 1 × MIC, filled blue circles; 0.5 × MIC, filled yellow circles). The buffer control is depicted with dark green cross symbols. The Y-axis displays the NPN fluorescence; the X-axis displays the time in minutes upon exposure. Average and standard deviations from three biological repeats are shown.

CecA is hypothesized to act as a wedge transferring the CBD and EAD moieties across the OM. The CBD subsequently binds the peptidoglycan layer, followed by cleavage through the EAD. Killing cells therefore requires outer membrane permeabilization. We determined whether equimolar amounts of 1D10, CecA-linker1-CBD, and CecA permeabilize the OM using 1-N-phenylnaphthylamine (NPN), as an established indicator for OM permeability. NPN exhibits fluorescence in a hydrophobic environment, but NPN cannot pass through the OM. When the integrity of the OM is compromised, NPN inserts into the hydrophobic lipid interior of the outer and inner membrane, which results in a strong increase in fluorescence (20). NPN fluorescence was evaluated under two different conditions: (i) cells resuspended in a dilute buffer (high intracellular turgor pressure) and (ii) cells resuspended in the same buffer supplemented with 150 mM NaCl (low intracellular turgor pressure). We observed that NPN fluorescence increases in a concentration-dependent manner for lysin 1D10, CecA, and CecA-linker1-CBD, indicating that they all effectively permeabilize the OM (Fig. 4). In the buffer containing 150 mM NaCl, fluorescence is lower, and the maximal values are observed later compared to cells resuspended in dilute buffer, indicating a reduced and slower OM permeabilization under these conditions. For those cells with low intracellular turgor pressure, CecA appears as the best OM permeabilizer, followed by lysin 1D10 and CecA-linker1-CBD. Consequently, outer membrane permeabilization, therefore, appears both protein and condition dependent.

### The inner membrane is effectively depolarized

**Fig 5:**
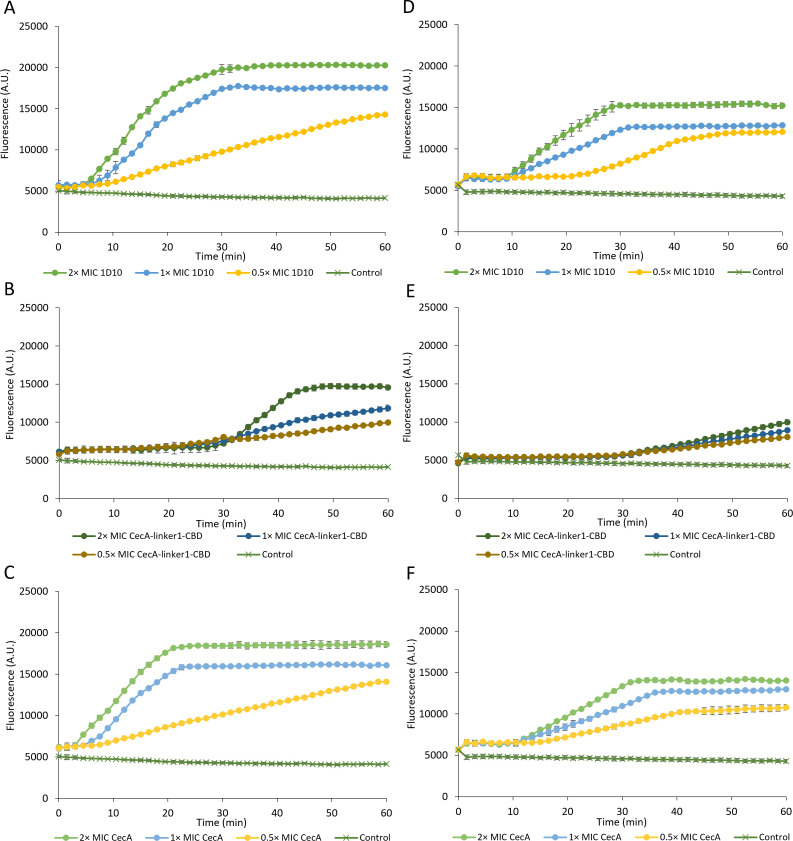
Measurement of inner membrane depolarization of 1D10 and derivatives. (A–C) Cells suspended in a dilute buffer (high intracellular turgor pressure). (D–F) Cells resuspended in the same buffer supplemented with 150 mM NaCl (low intracellular turgor pressure). Lysin 1D10 (**A and D**), CecA-linker-CBD (**B and E**), and CecA (**C and F**) are tested with equimolar concentrations, referring to the MIC of lysin 1D10 (2× MIC, filled light green circles; 1× MIC, filled blue circles; 0.5× MIC, filled yellow circles). The buffer control is depicted with dark green cross symbols. The Y-axis displays the DiSC_3_-5 fluorescence; the X-axis displays the time in minutes upon exposure. Average and standard deviations from three biological repeats are shown.

The mode of action of CecA is based on membrane depolarization. Therefore, we also quantified inner membrane depolarization of *A. baumannii* RUH134 induced by equimolar amounts of CecA, CecA-link1-CBD, and lysin 1D10 using DiSC_3_-5 as indicator. DiSC_3_-5 is quenched in the polarized inner membrane, but depolarization results in a fluorescent signal. The experiments were carried out under the same conditions as the NPN assay. Membrane depolarization takes place in a concentration-dependent manner for all three compounds (Fig. 5). Fluorescence is again more intense when cells are exposed to high intracellular turgor pressure. The highest fluorescence values were observed for cells with high intracellular turgor pressure (1D10 ≈ CecA > CecA-linker1-CBD). Under low intracellular turgor pressure, the onset of cellular permeabilization is delayed. These observations support that the intrinsic membrane depolarization capacity of CecA is maintained in the fusion proteins, although we cannot exclude that the other modules could theoretically affect membrane polarization as well.

### CecA and lysin 1D10 have comparable bactericidal activity

To compare the inhibitory and bactericidal effect, we determined the minimum inhibitory concentration (MIC) and minimum bactericidal concentration (MBC) for lysin 1D10, CecA, and CecA-linker1-CBD against four multidrug-resistant *A. baumannii* strains [three reference epidemiological clones of the European Union and the so-called Iraqibacter T-strain (21)] (Table 1). Notably, the MIC value equals the MBC for each combination, indicating that lysin 1D10, CecA, and CecA-linker1-CBD are all bactericidal. Both lysin 1D10 and CecA have a potent inhibitory and bactericidal activity against the tested *A. baumannii* strains, showing MIC/MBC values from 0.2 to 0.5 µM and 0.1 to 0.3 µM, respectively (Table 1). In the case of CecA-linker1-CBD, the MIC/MBC values against the *A. baumannii* strains increased approximately 10-fold compared to CecA. The additional modules (linker1-CBD) likely decrease the inhibitory and bactericidal activity. Reversion of this negative effect by the addition of the EAD in lysin 1D10 supports that CecA acts as a wedge to transfer the EAD across the OM, resulting in a local degradation of the peptidoglycan. The inhibitory and bactericidal activity of all compounds is marginally improved in the presence of 0.2 mM EDTA, which chelates stabilizing divalent cations from the OM. In all cases, the OM thus still limits the effects to some extent, as an additional OM destabilization by EDTA reinforces the inhibitory and bactericidal effect.

**TABLE 1:** MIC and MBC values of lysin 1D10, CecA, and 1D10 derivatives^*b*^ ^,^ ^*c*^

		MIC and MBC (µM)			
	Supplementation	*A. baumannii*			
		RUH875	RUH134	LUH5875	NCTC13423
Lysin 1D10^*a*^	Without EDTA	0.3	0.4	0.5	0.2
	0.2 mM EDTA	0.2	0.1	0.2	0.1
CecA	Without EDTA	0.2	0.2	0.2	0.3
	0.2 mM EDTA	0.1	0.1	0.1	0.1
CecA-linker1-CBD	Without EDTA	1.7	1.9	1.9	1.9
	0.2 mM EDTA	1.4	1.1	1.1	1.7
CecA-linker1-EAD	Without EDTA	0.5	0.5	0.6	0.3
	0.2 mM EDTA	0.3	0.2	0.3	0.2
CecA-CBD-EAD	Without EDTA	NA	NA	NA	NA
	0.2 mM EDTA	NA	NA	NA	NA
CBD-EAD	Without EDTA	NA	NA	NA	NA
	0.2 mM EDTA	NA	NA	NA	NA
CecA-linker2-CBD-EAD	Without EDTA	0.5	0.5	0.5	0.3
	0.2 mM EDTA	0.3	0.2	0.3	0.2
CecA-linker3-CBD-EAD	Without EDTA	NA	NA	NA	NA
	0.2 mM EDTA	NA	NA	NA	NA
CecA-linker4-CBD-EAD	Without EDTA	0.2	0.4	0.3	0.1
	0.2 mM EDTA	0.1	0.1	0.1	0.1
CecA-linker5-CBD-EAD	Without EDTA	0.5	0.3	0.3	0.2
	0.2 mM EDTA	0.3	0.1	0.1	0.2
CecA-linker6-CBD-EAD	Without EDTA	0.7	0.5	0.5	0.6
	0.2 mM EDTA	0.4	0.3	0.3	0.3
CecA-linker7-CBD-EAD	Without EDTA	0.4	0.3	0.4	0.2
	0.2 mM EDTA	0.3	0.2	0.2	0.1

^*a*^ Data published previously, but shown for comparison (10).

^*b*^ NA: no activity was seen up to 4 µM.

^*c*^ All experiments were done in Mueller-Hinton medium without or supplemented with 0.2 mM EDTA (final concentration). Data represent the mode of three biological replicates. Lysin 1D10 consists of (i) the OMP peptide CecA; (ii) a neutral, short, and flexible linker; (iii) the modified CBD; and (iv) an EAD. All other constructs lack one or more parts of 1D10. MIC and MBC values are the same for all strains.

### Dissection of lysin 1D10 demonstrates the necessity of the linker for the killing mechanism, whereas the CBD is not essential

To gain a better understanding on the contribution of the different modules to the mode of action of 1D10, three additional derivatives were constructed using VersaTile (10). CecA-linker1-EAD and CecA-CBD-EAD (Fig. 1) were constructed to elucidate the role of the CBD and linker1, respectively, whereas CBD-EAD served as a negative control (Table S1).

We determined the MIC and MBC values of all additional derivatives against the same four multidrug-resistant *A. baumannii* strains (Table 1). CBD-EAD does not display any inhibitory activity up to 4 µM, indicating that this protein is not able to pass the OM without the help of CecA. Also, CecA-CBD-EAD becomes inactive, with no inhibitory activity up to 4 µM. CecA-linker1-EAD retains its inhibitory and bactericidal activity (0.3–0.6 µM), even when the CBD is absent (Table 1). The MIC value still equals the MBC value, indicating that CecA-linker1-EAD remains bactericidal. As observed previously, its antibacterial activity increases when 0.2 mM EDTA is added. We conclude that the linker is essential to maintain each of both killing mechanisms. Without this linker, the inhibitory activity is completely abolished, including when the OM is destabilized by low concentrations of EDTA. In addition, CecA appears to no longer function as a wedge to transfer the EAD moiety. Conversely, the CBD is not essential, since CecA-linker1-EAD retains its activity.

### While the linker modulates antibacterial activity, not every linker is functional

We further focused on the essential role of the linker and exchanged the original, flexible linker1 by six linkers with different predicted properties (Table S2). Specifically, they differ in predicted length, flexibility, and secondary structure. The MIC and MBC values were determined for all six constructs against the four *A. baumannii* strains (Table 1). CecA-linker3-CBD-EAD does not retain any inhibitory activity against the four *A. baumannii* strains. The activity of CecA-linker6-CBD-EAD is twofold to threefold lower, depending on the strain. On the other hand, variants with linkers 2, 4, 5, or 7 have a similar or slightly improved inhibitory and bactericidal activity compared to 1D10 (Table 1). Again, all MBC values are identical to the MIC values, and the antibacterial activity is increased when the cells are further sensitized with 0.2 mM EDTA. These results demonstrate that the linker affects the antibacterial activity to variable extent. No correlation is observed between the predicted properties of the linker and the eventual activity. Both predicted flexible and rigid linkers can be effective. For example, linker 3, which eliminates all activity, forms a predicted rigid helix, but other linkers with a rigid helix (linker 4, linker 7) remain functional.

### *Acinetobacter baumannii* is the most susceptible species for both lysin 1D10 and CecA

We compared the antibacterial spectrum of lysin 1D10 and CecA against a panel of Gram-positive and Gram-negative bacteria using MIC/MBC assays (Table S3). CecA has a broad antibacterial spectrum when 0.2 mM EDTA is included and is active against Gram-positive and Gram-negative bacteria with MIC values between 0.1 and 2.2 µM. *Enterococcus faecalis* HC-1909-5 is a notable exception. When EDTA is omitted, the spectrum is more confined, and CecA shows residual activity against *Klebsiella pneumoniae*, *Pseudomonas aeruginosa* Br667, *Escherichia coli*, *Salmonella enteritidis*, *Salmonella* Typhimurium, *Shigella flexneri*, vancomycin-resistant *Enterococcus*, and *Staphylococcus aureus* with MIC values ranging from 1.1 to 8.7 µM. The antibacterial spectrum of 1D10 (<1.4 µM) is restricted to *A. baumannii*, *E. coli*, *S*. Typhimurium, *S. enteritidis*, and *S. flexneri* ranging from 0.1 to 1.1 µM when EDTA is included. In the absence of EDTA, no antibacterial activity was observed against other species than *A. baumannii* up to at least 1.4 µM for lysin 1D10. Altogether, these data show that *A. baumannii* is the most susceptible species for both CecA and lysin 1D10, but when cells are sensitized, CecA can inhibit a broader panel of species.

The combination of different antibiotics (amikacin, imipenem, and colistin) with lysin 1D10 against *A. baumannii* and *P. aeruginosa* was ascertained using checkerboard assays (Fig. S6). In the case of *A. baumannii*, an additive effect with fractional inhibitory concentrations indices (FICI) >0.5 and ≤4 was found for all three antibiotics in combination with lysin 1D10. Interestingly, the combination of lysin 1D10 with colistin is synergistic against *P. aeruginosa* strains. An amount of 0.5 µM lysin 1D10 reduces the colistin MIC fourfold to eightfold. As such, colistin sensitizes *P. aeruginosa* strains to lysin 1D10 (or vice versa).

### Lysin 1D10 is a mesophilic but thermoresistant protein with high refolding capacity

Proteinaceous antimicrobials such as CecA and lysin 1D10 may suffer from a limited thermal stability. The thermoresistance of CecA and 1D10 was therefore evaluated by exposing the compounds to a range of different temperatures (Tms) (up to 90°C for 30 min) and sterilization conditions (121°C, 210 kPa for 20 min) (Table S4), followed by MIC and MBC determination against *A. baumannii* NCTC13423. In all cases, the MIC values remain identical to the MBC values. Interestingly, lysin 1D10 exhibits a remarkably high thermoresistance, while its MIC/MBC values do not change, even after exposure to sterilization conditions. In contrast, CecA lost its antibacterial activity at the tested temperatures above 70°C. To determine whether a specific module or interaction between modules is responsible for the observed thermoresistance of lysin 1D10, the thermoresistance of CecA-linker1-CBD and CecA-linker1-EAD was evaluated individually using the same set-up. CecA-linker1-CBD is inactivated at temperatures over 60°C, while CecA-linker1-EAD shows the same thermoresistance as 1D10. To elucidate the role of the linker, we tested the constructs with different linkers that showed the same or an improved antibacterial activity compared to 1D10. The constructs with linker 4, 5, or 7 show similar thermoresistance as 1D10, whereas the constructs with linkers 2 and 6 are inactivated at 90°C and 80°C, respectively. In spite of this high thermoresistance, the unfolding temperature of 1D10 was measured at 57°C ± 2°C by differential scanning fluorimetry using SYPRO Orange (Fig. S7). After unfolding and refolding during this experiment, the 1D10 sample was recovered, and again, an unchanged MIC was observed. Altogether, these data indicate that 1D10 is a mesophilic protein that does not irreversibly denature upon unfolding. Once the temperature is lowered, the protein refolds, thereby regaining its antibacterial activity. The truncation analysis indicates that the EAD in 1D10 is most likely responsible for this behavior. By contrast, CecA is less thermoresistant than 1D10.

### Lysin 1D10 and its derivatives do not trigger cytotoxicity on a HaCaT epithelial cell line

**Fig 6:**
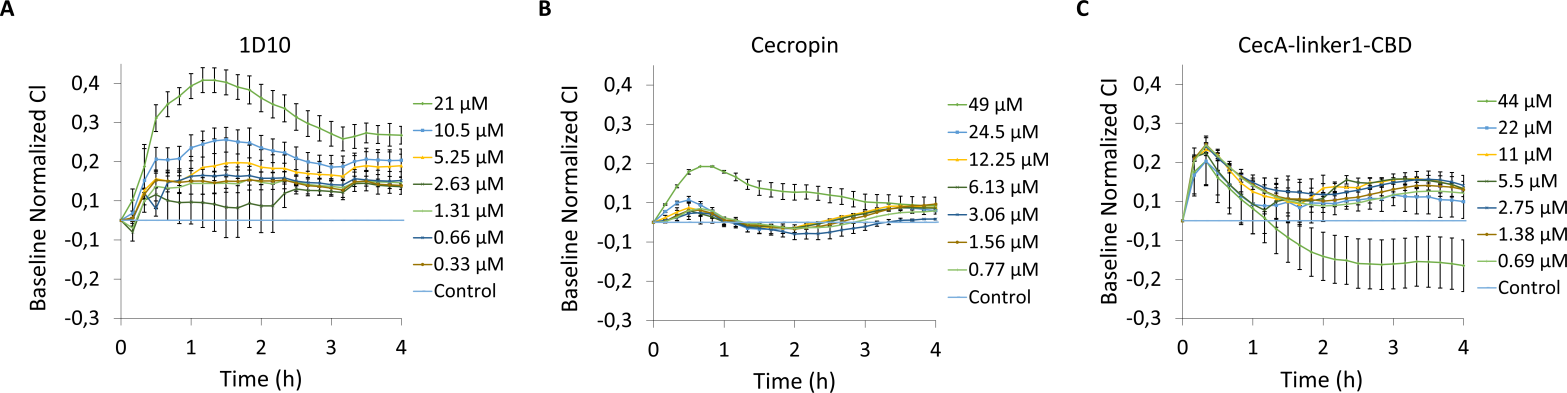
Cytotoxicity. Variation in the normalized cell index (CI) of HaCaT monolayers treated with different concentration of (**A**) 1D10, (**B**) cecropin, and (C) CecA-linker1-CBD is shown. Normalization of data was performed at 10 min after protein addition, with respect to the CI observed in the control sample (value 0 in the graph). Values represent average ± standard deviation of three replicates.

Antimicrobial peptides are frequently associated with cellular toxicity. To determine the potential toxicity of 1D10, CecA-linker1-CBD and CecA, an experiment was performed by exposing the human epithelial cell line, HaCaT, to a range of protein concentrations up to 20 µM for 1D10 and up to 45 µM for CecA-linker1-CBD and CecA (Fig. 6).

None of the proteins tested induce cytotoxicity on the epithelial cell line, since the values of the baseline normalized cell index (CI) do not decrease. An exception is the highest concentration of CecA-linker1-CBD (44 µM), where a small decrease in the normalized CI can be observed (Fig. 6B). In contrast, an increase in this index is observed with a maximum level reached after 1 h and 30 min for 1D10 and 40 min for CecA-linker1-CBD and CecA (Fig. 6A, B, and C, respectively). This behavior suggests temporal modifications in cellular receptors such as G-protein coupled receptors, among others (22). This effect is concentration dependent, particularly in the case of 1D10. The lack of cytotoxic effects was confirmed by confocal laser microscopy images obtained after treatment with the proteins for 40 min and 4 h (Fig. S8 and S9, respectively), showing no observable morphological changes. Therefore, we conclude that 1D10, CecA-linker1-CBD, and CecA are not cytotoxic toward the human epithelial cell line, HaCaT.

## DISCUSSION

The engineered lysin 1D10 was previously identified as a variant active against *A. baumannii* in elevated human serum concentrations. Only a minority of the screened variants was active in human serum, and only 1D10 was active against all *A. baumannii* strains tested under these conditions (10). Therefore, we focused on better understanding the mode of action of 1D10.

Time-lapse series of previously engineered lysins with OMP peptides [LoGT-008 (6); Art-175 (12)] showed a sudden and complete lysis of *Pseudomonas aeruginosa* cells after exposure to the lysin. Defraine et al. (13) demonstrated that this mode of action is driven by the high intracellular turgor pressure, which cannot longer be contained after the disintegration of the cell wall (13). These observations were microscopically similar to observations made with Gram-positive pathogens exposed to specific lysins (23, 24) with the only difference that the latter do not need an OMP peptide to reach the peptidoglycan layer. Time-lapse microscopy showed a surprisingly distinct mode of action for lysin 1D10 compared to previously reported lysins engineered with an outer membrane permeabilizing peptide. Instead of inducing full osmotic lysis, 1D10 creates local cell wall bulging at the septa or poles, exacerbated by a high turgor pressure. In addition, an apparent redistribution of the cellular content takes place.

Integrating the obtained observations, the killing mechanism of lysin 1D10 can best be explained by a translocation of the EAD across the OM by a local CecA-dependent OM permeabilization. The EAD then locally cleaves the peptidoglycan layer, resulting in cell wall bulging. The localized and more focused outer membrane penetration observed for lysin 1D10 (compared to previously reported engineered lysins that induce full osmotic lysis) may be explained by the preference of CecA to attack dividing cells at the septum or non-dividing cells at the new pole (14). Indeed, the curved septal region is enriched in cardiolipin, a phospholipid with four fatty acids that can accommodate the high curvature, and a negatively charged lipid head group. Cardiolipin may act as a potential docking site for CecA. Single-cell fluorescence microscopy analysis revealed that CecA (from the moth *Hyalophora cecropia*) acts on dividing and non-dividing *E. coli* cells after 1 and 2–8 min, respectively. Subsequent cytoplasmic membrane permeabilization occurs approximately 30 s later (14). This rate and order are comparable to our observations.

Our data suggest that the membrane depolarizing activity of CecA may also remain active within the fusion protein and partially contribute to the overall antibacterial activity of lysin 1D10. This would be surprising as the large cargo fused to CecA is expected to interfere with its biological function of membrane destabilization. Nevertheless, the NPN and DiSC_3_-5 assays show that intrinsic OM permeabilization and inner membrane depolarization take place even when a cargo is fused to the peptide. Specifically, the intrinsic OM permeabilizing capacity of CecA is only marginally inhibited by the additional modules linker1-CBD. This inhibition is partially reverted when linker1-CBD-EAD is fused. The latter may be explained by the high net positive charge of the EAD (pI 8.12) in lysin 1D10 which may enhance the OM permeabilization capacity compared to CecA-linker1-CBD or by EAD-induced cell wall disintegration. The limited outer membrane penetration of lysin 1D10 targeted to the septum and poles only and the consequent local cell wall bulging instead of full osmotic lysis may allow sufficient time for CecA to exert its bactericidal effect by inducing membrane depolarization. This is in contrast to other engineered lysins which penetrate the outer membrane across the entire surface. This results in a rapid full osmotic lysis, masking or preceding a potential effect of the OMP peptide. With the currently available data, it cannot be concluded whether both peptidoglycan degradation and membrane depolarization are essential for killing or whether they are complimentary/mutually exclusive. Mutagenesis studies on the unknown active residue(s) of the EAD may provide further confirmation. A BLAST search indicated 8-amino-acid sugar-binding sites. However, none of them had the typical active site seen in either glycosidases or lytic transglycosylases.

The obvious influence of the intracellular turgor pressure is logically explained by the need for sufficient pressure to lyse the cells because an OM alone can also stabilize cell wall-deficient cells when the pressure is not too high. Such cell wall-deficient cells can recover an intact peptidoglycan sacculus when growing on nutrient-rich media, surviving temporal cell wall deficiency (25). It is therefore expected that peptidoglycan needs to be degraded more extensively under conditions of low intracellular turgor pressure. Additionally, the lower OM permeabilizing capacity of CecA, CecA-linker1-CBD, and lysin 1D10 when cells have a lower intracellular pressure (Fig. 4) may also contribute to the reduced antibacterial activity under these conditions. This is consistent with the reduced membrane depolarization under the same conditions, indicating that a lower number of molecules effectively pass the OM (Fig. 5). Nevertheless, the killing effect of the osmotic shock supports that the cell wall integrity has been weakened by the EAD.

These experiments led to an improved insight in the mode of action of lysin 1D10 but do not explain why this variant excels in activity under elevated human serum conditions. As shown here, CecA is also active in human serum. However, not every fusion of CecA and the EAD is functional. A direct fusion is inactive, and not every linker provides the right configuration to result in an antibacterial lysin. Linker engineering has been proven to be a possible successful approach to improve the antibacterial properties of a lysin (26), but here, we only achieved a slight improvement of the MIC/MBC values for CecA-linker4-CBD-EAD. Interestingly, the CBD seems to be not essential in 1D10 particularly since CecA-linker1-EAD has a similar MIC/MBC as 1D10. A few reasons could explain this observation. First, the EAD is the C-terminal domain of the virion-associated lysin gp16 of phage BcepC6B and therefore naturally functions without a CBD to perform its activity. In addition, the high net positive charge of the EAD (pI 8.12) could result in a natural attraction of the EAD toward the cell wall, excluding the need of a CBD.

We conclude that empirical high-throughput engineering remains essential to identify the lysin variant with desired properties and that different mode of actions can occur depending on the modular composition, implying that a careful design and knowledge of the building blocks are pivotal. This process can be facilitated by a high-throughput assembly method such as VersaTile (10). Particularly in this study, the availability of all building blocks (“tiles”) enabled the rapid construction of variants with different modular compositions using VersaTile.

In a final step, the antibacterial spectrum, thermoresistance, and cytotoxicity of CecA and lysin 1D10 were compared. Antimicrobial peptides often show a broad antibacterial activity, which is not always desired (11, 27). Here, the antibacterial spectrum of CecA was similar to the spectrum of lysin 1D10. However, permeabilization of the outer membrane with EDTA showed that a broad number of species are more susceptible for CecA. It can be concluded that lysin 1D10 has a more focused spectrum, in which *A. baumannii* is by far the most susceptible species. Lysin 1D10 was found to be highly thermoresistant, as MIC and MBC values were fully retained, even after sterilization conditions. A truncation analysis indicates that the EAD is important for this observed thermoresistance. A high thermoresistance has been observed before for virion-associated lysin domains, e.g., KMV36C retains 26% of its activity after 2 h at 100°C and 21% after sterilization conditions (28), a feature attributed to rapid refolding after heat treatment (29). This is also the case for lysin 1D10 as the protein has a Tm of 57°C ± 2°C. Other endolysins have been reported to be thermostable up to 90°C and even higher (30 – 34). CecA itself is only stable up to 70°C, similar to many other antimicrobial peptides (35). Therefore, fusion to the lysin appears to enhance its stability within lysin 1D10. In terms of cytotoxicity, no differences could be observed for CecA and lysin 1D10 at relevant concentrations when using the human epithelial cell line, HaCaT. Nevertheless, some studies report selective cytotoxicity of cecropins towards certain human cell lines, indicating possible cell line dependency. This is also the case for cecropins that are fused to other peptides (36, 37).

In summary, this study highlights the importance of understanding the killing mechanism of engineered lysins targeting Gram-negative bacteria. More detailed insights will eventually result in smarter engineering approaches and designer rules that steer the rational engineering process.

## MATERIALS AND METHODS

### Bacterial strains and growth media

All *A. baumannii* strains (38) were routinely grown at 30°C in Luria-Bertani (LB) broth (1% tryptone, 0.5% yeast extract, 1% NaCl) with shaking (200 rpm) or on LB supplemented with 1.5% of agar. *K. pneumoniae* ATCC13883, *K. oxytoca* 1, *P. aeruginosa* PA14, *P. aeruginosa* PAO1, *P. aeruginosa* Br667, *E. coli* ETEC1 O:149, *E. coli* (APEC)CH2, *S. flexneri* LMG10472, *Bacillus subtilis* sp. *subtilis*, *S. aureus* VISA/HIP5827, *S. aureus* VRS1/HIP11714, *B. cereus* LMG9610, *S*. Typhimurium LT2, and *S. enteritidis* ATCC13076 were grown in the same conditions but at 37°C. *E. faecalis* HC-1909-5, *Enterococcus* VRE6, and *E. faecium* VR-1802-13 were grown in brain heart infusion (BHI) broth (Lab M, United Kingdom) or agar at 37°C. *S. aureus* subsp. *aureus* Rosenbach; ATCC6538 was grown in Mueller-Hinton (MH) broth (Becton Dickinson, Belgium) or agar at 37°C. *E. coli* TOP10 (Agilent Technologies, Belgium) was used for plasmid storage and *E. coli* BL21(DE3)-RIL (Agilent Technologies, Belgium) for the expression of all constructs. These cultures were grown at 37°C on LB supplemented with 100 µg/mL ampicillin or 50 µg/mL kanamycin and 5% (wt/vol) sucrose.

### Creation of lysin 1D10 derivatives

The VersaTile technique was used to generate all constructs mentioned in this study. Basically, VersaTile is a DNA assembly technique to combine building blocks (“tiles”). A tile comprises a coding sequence flanked by position tags that label the final position in the assembled DNA molecule. Some tiles were previously constructed [CecA, linker1, CBD, and EAD at positions 1, 2, 3, and 4, respectively (10)]. New tiles were constructed for predicted linkers at position 2; for the CBD, at position 1 and 2; and for the EAD, at positions 2 and 3. HexaHis-coding tags to complete empty positions were also constructed. Predicted linker tiles were constructed by cassette hybridization of primers (Integrated DNA Technologies, Leuven, Belgium) as described in reference (10). Briefly, both primers (Table S5) were mixed in equal ratio (5 µM each), heated for 5 min at 95°C, and allowed to cool down overnight. To generate the EAD and CBD tiles of lysin 1D10 at new positions, primers were constructed that have extended 5′ sequences, which contain the new position tags. A standard PCR with these primers was done, using the original tiles of the EAD and CBD as template. Amplification was done using Phusion High-Fidelity DNA polymerase (Thermo Fischer Scientific, Belgium) following the manufacturer’s instructions with 1-ng DNA as template. The resulting primer cassettes or amplicons were ligated into entry vector pVTE using SapI (Thermo Fischer Scientific, Belgium) and T4 DNA ligase (Thermo Fisher Scientific, Belgium). *E. coli* TOP10 was transformed with the ligation mixtures. Correct clones were verified by Sanger sequencing (LGC genomics, Germany). The assemblies were prepared as described before (10). Briefly, a tile mixture was made by taking 1 µL from each tile (50 ng/µL). Next, the VersaTile assembly reaction mixture was made containing the following components: 1 µL of 100 ng/µL pVTD, 4 µL tile mixture, 1 µL BsaI (10 U/µL), 3 µL T4 DNA ligase (1 U/µL), and 2 µL T4 DNA ligation. Ultrapure water was added to the mixture to reach a total reaction volume of 20 µL. Assembly was then performed in a PCR device with the following parameters: 50 cycles of 37°C for 2 min followed by 16°C for 3 min; a cycle of 5 min at 50°C; and finally, 5 min at 80°C. *E. coli* TOP10 chemical competent cells were transformed with the assembly mixtures. After sequencing confirmation, *E. coli* BL21(DE3)-RIL was transformed with the validated plasmids.

### Protein expression and purification of lysin 1D10 and its derivatives

Expression and purification were done as previously described (12). *E. coli* BL21(DE3)-RIL cells, containing the construct of interest, were freshly plated on LB 1.5% agar, supplemented with 50 µg/mL kanamycin and 5% (wt/vol) sucrose. Five colonies were inoculated in 20-mL LB medium supplemented with 50 µg/mL kanamycin and incubated overnight in an orbital shaker (37°C, 200 rpm). This overnight culture was used to inoculate 0.5-L LB medium, followed by growth at 37°C until OD_600_ of 0.5–0.6. Expression was induced by adding 1 mM of IPTG, and the culture was placed at 16°C for 18 h at 200 rpm. Lysis of the cells was done by dissolving the pellet in 20 mL lysis buffer (20 mM NaH_2_PO_4_/Na_2_HPO_4_ 0.5 M NaCl 50 mM imidazole pH 7.4) followed by three freeze-thawing steps and sonication (Q125, Qsonica). The soluble fraction was obtained by centrifugation at 16,000 × *g* for 20 min and filtration through 0.22-µm PES filter (Novolab, Belgium). The soluble protein fraction was purified with His GraviTrap columns (GE Healthcare, Belgium) according to the manufacturer’s instructions. Purified proteins were stored at 4°C in elution buffer (20 mM NaH_2_PO_4_/Na_2_HPO_4_ 0.5 M NaCl 500 mM imidazole pH 7.4). Dialysis was performed using Slide-A-Lyzer MINI Dialysis Devices (Thermo Fischer Scientific, Belgium) against 20 mM HEPES-NaOH with 500 mM NaCl (pH 7.4). Depending on the experiment, a subsequent dialysis was performed against 20 mM HEPES-NaOH with 150 mM NaCl (pH 7.4) and thereafter against 20 mM HEPES-NaOH (pH 7.4). Protein concentrations were determined by spectrophotometry using a DS-11 spectrophotometer (Denovix, Wilmington, USA).

### Chemical synthesis of CecA

CecA (sequence: GGLKKLGKKLEGAGKRVFNAAEKALPVVAGAKALRK) was synthesized by GenScript (Leiden, Netherlands) by solid-phase synthesis and purified by reversed-phase chromatography. The sequence of the peptide was confirmed by liquid chromatography-mass spectrometry, and solubility in water was confirmed visually. CecA was diluted in sterile distilled water, aliquoted, and stored at −20°C. For some experiments, an aliquot was thawed and diluted in 20 mM HEPES-NaOH and 500 mM NaCl (pH 7.4) or 20 mM HEPES-NaOH and 150 mM NaCl (pH 7.4).

### Time-lapse microscopy

Time-lapse microscopy was performed as described by Defraine et al. (13), with minor modifications. Two type of pads were used: (i) an agar pad made with 20 mM HEPES-NaOH pH 7.4; (ii) a serum pad made with complement deactivated human serum. For technical reasons, it was not possible to create 100% human serum pads. The reason is the high abundance of proteins in human serum that precipitate upon heating the mixture to dissolve the agar. Therefore, the human serum needed to be diluted to 60% before clear pads could be made. The dilution was made with 20 mM HEPES-NaOH 150 mM NaCl pH 7.4 to ensure low turgor pressure. The experiment starts by inoculating fresh MH with an overnight culture of *A. baumannii* RUH134 (20× diluted) and grown at 30°C up to OD_600_ of 0.6. To achieve a high intracellular turgor pressure, the culture was washed three times with 20 mM HEPES-NaOH pH 7.4. When a low intracellular turgor pressure was needed, the culture was washed three times with 100% (complement deactivated) human serum. Cells were kept on ice until use. Lysin 1D10 or its derivatives were dialyzed before use against either 20 mM HEPES-NaOH pH 7.4 (high turgor pressure) or 20 mM HEPES-NaOH 150 mM NaCl pH 7.4 (low turgor pressure). CecA was diluted in the same buffers. Immediately before the start of the experiment, CecA, lysin 1D10, or the derivatives were added to the bacterial cells to achieve a final concentration of 2 µM, while not diluting the human serum more than 10%. For imaging, the bacterial cells were placed on a pad, with a cover glass attached on top with the help of Gene Frames (Life Technologies) as described in reference (39). Images were acquired using a temperature controlled (Okolab Ottaviano, Italy) Ti-Eclipse inverted microscope (Nikon, Champigny-sur-Marne, France) equipped with a TI-CT-E motorized condenser and a Nikon DS-Qi2 camera using NIS-Elements (Nikon) (36). All images were equally processed using NIS Elements viewer (version 5.21.00, Nikon) and ImageJ (version 1.52p). Microscopic fields covering approximately 100–200 *A*. *baumannii* cells were analyzed for each condition. Events were differentiated for cells both under high and low turgor pressure, undergoing cell wall bulging either at the poles or at the septum or reorganization of the intracellular content (cell wall bulging was always associated with reorganization of the intracellular content; this is indicated in the graphs as cell wall bulging and reorganization of the intracellular content). If the cells did not show any of these events, they were classified as “No difference.”

### Muralytic activity assay

EnzChek Lysozyme Assay Kit (Invitrogen) was used to measure peptidoglycan degradation activity according to the instructions of the manufacturer (0.1 M sodium phosphate buffer, 0.1 M NaCl, pH 7.5, 2 mM sodium azide). Fluorescence was recorded in a TECAN Infinite 200 PRO reader. A fluorescein concentration gradient was used to standardize the results based on a minimum (a blank of untreated substrate) and a maximum read (maximum fluorescence detected for fluorescein). The calibration curve to calculate specific activities was obtained by applying linear regression on the fluorescence measurements for a concentration gradient of a sample with a known activity, supplied with the kit. Two independent replicates were conducted with two technical replicates each.

### Outer membrane permeabilization assay

The experiment was carried out as described in reference (40). Briefly, permeabilization of the bacterial OM was monitored by the uptake of fluorescent 1-N-phenylnaphthylamine (NPN, Acros, Belgium). *A. baumannii* RUH134 was grown for 16 h in MH at 30°C in an orbital shaker at 200 rpm. The next day, fresh MH was inoculated, and cells were grown to OD_600_ of 0.6. The culture was washed and resuspended to 0.25 at OD_600_ in 50 mM HEPES-NaOH pH 7.4 with or without 150 mM NaCl. NPN was added to the cell suspension to a final concentration of 10 µM. Next, 50 µL of 1D10, CecA-linker1-CBD, or CecA in equimolar concentration (0.5×, 1×, or 2× MIC, referring to the MIC of lysin 1D10, 0.4 µM or 12 µg/mL) was added to the mixture in a 96-well plate. The fluorescence intensity was measured each 90 s using a Tecan fluorescence MTP reader (excitation: 350 nm, emission: 420 nm).

### Cytoplasmic membrane depolarization assay

The experiment was carried out as described in reference (40). Briefly, depolarization of the cytoplasmic membrane was measured by the release of the potential-sensitive dye DiSC_3_-5 (Sigma-Aldrich, Belgium). *A. baumannii* RUH134 was grown for 16 h in MH at 30°C in an orbital shaker at 200 rpm. The next day, fresh MH broth was inoculated and grown to OD_600_ of 0.6. The culture was washed three times with 50 mM HEPES-NaOH pH 7.4 containing 20 mM glucose. The bacteria were resuspended to 0.05 at OD_600_ in 50 mM HEPES-NaOH, 20 mM glucose, 100 mM KCl, and with or without 150 mM NaCl (pH 7.4). DiSC_3_-5 was added to achieve a final concentration of 1 µM, and its fluorescence was stabilized for 1 h. Next, equimolar concentrations of 1D10, CecA-linker1-CBD, or CecA (0.5×, 1×, or 2× MIC) were added. The fluorescence intensity was measured each 90 s using a Tecan fluorescence MTP reader (excitation: 622 nm, emission: 670 nm).

### Killing assay in buffer and human serum

The killing assay has been performed as described previously (10) with minor modifications. Lysin 1D10 or derivatives thereof were dialyzed against 50-µL 20 mM HEPES-NaOH 150 mM NaCl pH 7.4. The peptide was diluted in the same buffer. Briefly, *A. baumannii* RUH134 was grown in LB to OD_600_ of 0.6. Thereafter, the culture is washed three times in 20 mM HEPES-NaOH pH 7.4 and diluted to ~10^6^ CFU/mL in 20 mM HEPES-NaOH pH 7.4 or 100% human serum (MP Biomedicals, Belgium). The complement in the human serum was heat inactivated (56°C for 30 min). A cell suspension (270 µL) was mixed with 15 µL of 30 µM dialyzed 1D10 (or derivative) (final concentration 1.5 µM) and 15 µL 20 mM HEPES-NaOH pH 7.4 with or without 4 mM EDTA (final concentration 0.2 mM EDTA). Samples containing serum, therefore, have a final serum concentration of 90%. After incubation for 1 h at room temperature, cell dilutions were made in 20 mM HEPES-NaOH pH 7.4 or 100% complement inactivated human serum and plated in triplicate. Next, plates were incubated overnight at 30°C. The log reduction was quantified as the relative inactivation level in log units [log_10_(N_0_/N_i_) with N_0_ the initial number of untreated cells and N_i_ the number of residual cells after treatment].

### Minimum inhibitory and bactericidal concentration assay

All bacteria were grown overnight in MH broth at 30°C or 37°C in an orbital shaker at 200 rpm. The overnight culture was used to inoculate fresh MH broth in a 1:10 ratio, and the culture was grown to OD_600_ of 0.6. Next, the culture was diluted to 2 × 10^5^ CFU/mL in MH broth. CecA or protein (20 mM HEPES-NaOH 150 mM NaCl pH 7.4) was added to the cell suspension with a final concentration between 0 and 5.5 µM in 0.5 µM steps, with and without 0.2 mM EDTA in a 96-well plate. If no inhibitory activity was observed, the assay was repeated with a final concentration between 0 and 10.9 µM in 1.1 µM steps, respectively, with and without 0.2 mM EDTA, in a 96-well plate. Controls included the bacteria without the peptide/protein, without EDTA (positive control) and not inoculated broth (negative control). The plate was incubated for 18 h at 30°C or 37°C, and the MIC was determined as the lowest concentration that gave complete growth inhibition. The MBC was determined by dropping 5 µL of each well of the MIC assay on MH agar. The plate was incubated for 18 h at 30°C or 37°C, after which the MBC was determined as the lowest concentration that yielded no growth.

### Checkerboard assay

MIC values of 1D10 (0–2.2 µM), colistin (0–7 µM), imipenem (0–214 µM), and amikacin (0–874 µM) were determined in MH broth following a conventional microdilution assay as described above. This assay was performed against exponentially grown *A. baumannii* strains (RUH134, NCTC13423) and *P. aeruginosa* strains (Br667, PA14, PAO1) to final density of 10^6^ CFU/mL. Based on the determined MIC values, the interaction of both 1D10 (0–2.2 µM) and each of the four antibiotics (1/64× MIC–2× MIC) was evaluated using a checkerboard assay. After 18-h incubation at 37°C, MIC values for each drug were determined independently and in combinations. In addition, fractional inhibitory concentrations (FIC) for each combination were calculated as MIC of drug A in presence of drug B divided by MIC of drug A alone. Then, FICI was calculated as sum of FIC values for both drugs. The interaction is interpreted as synergy, additive or antagonistic if FICI ≤0.5, 0.5 < FICI ≤ 4, or FICI >4, respectively.

### Thermoresistance assay

A volume of 500 µL of 1.4 µM 1D10, 2.8 µM CecA-linker1-CBD, 2.0 µM CecA-linker1-EAD or CecA (10.9 µM) were incubated at different temperatures in 20 mM HEPES-NaOH 150 mM NaCl pH 7.4. The proteins were exposed for 30 min to a temperature between 30°C and 90°C in steps of 10°C or a standard sterilization conditions (20 min at 121°C with a pressure of 210 kPa). The proteins were immediately placed on ice for 10 min after incubation at each temperature. Subsequently, the MIC and MBC were determined as described previously.

### Thermostability and Tm calculation

Thermal unfolding of 1D10 was monitored by recording SYPRO Orange (Thermo Fischer Scientific; Waltham, MA, USA) fluorescence on the UNcle platform (Unchained Labs; Pleasanton, CA, USA) according to the manufacturer’s instructions. Briefly, a sample of 9 µL at a protein concentration of 0.7 mg/mL, containing SYPRO Orange at a 15× concentration, was loaded in triplicate in capillaries and sealed afterward. Subsequently, the samples were heated from 25°C to 95°C at a heating rate of 0.3°C/min. As a control, the blank buffer (20 mM HEPES-NaOH, 500 mM NaCl, pH 7.4) was used. Afterward, the samples were recovered, and an MIC assay was carried out to verify the antibacterial activity. The melting Tm was calculated from three technical repeats from the first derivative of the area per nanometer values using the built-in tools of the UNcle platform.

### Epithelial cell line HaCaT and culture conditions

All media and reagents were purchased from Sigma-Aldrich. HaCaT cells were cultured in DMEM containing heat-inactivated FBS (10%, vol/vol), 4.5 g/L glucose, 2 mM L-glutamine, and a mixture of antibiotics (50 µg/mL of penicillin, streptomycin, and gentamicin) and antifungicide (1.25 µg/mL amphotericin B) at 37°C, 5% CO_2_ atmosphere in a CO_2_-Series Shel-Lab incubator (Sheldon Manufacturing, Inc., OR, USA). Maintenance of the cell line was performed two times per week. When cells reached approximately 90% confluence, they were harvested with TrypLE Express solution and were used to carry out the further assays.

### Monitoring the behavior of HaCaT in real time in the presence of the peptide/proteins

The real-time cell analyzer (RTCA-DP) xCelligence (ACEA Bioscience, Inc., San Diego, CA, USA) was used to monitor in real time the growth of the cell line and the cytotoxic effect observed when adding the different proteins as previously described (37). Briefly, the RTCA equipment was introduced into a Heracell-240 Incubator (Thermo Electron LDD GmbH, Langenselbold, Germany) at 37°C with 5% CO_2_ atmosphere. A HaCaT cell suspension of 10^5^ cells/mL was made in DMEM, and 100 µL was added to each well of 16-well E-plates. The plates were connected to the holders in the equipment and incubated at 37°C with 5% CO_2_ to record the CI (impedance measurement) every 10 min for 72 h in total. When the cell index reached the confluent phase, indicating formation of a monolayer of keratinocytes (after 20 h of incubation), different concentrations of the peptide/proteins were made by twofold dilutions in DMEM, and 100 µL was added. The concentration ranges were 21 to 0.3 µM for 1D10, 44 to 0.69 µM for CecA-linker1-CBD, and 49 to 0.7 µM for CecA. Each condition was tested in triplicate. As a control, 100 µL of DMEM was used. The cell index was recorded for additional 50 h to monitor any change in the cell line. Data normalization was performed as previously described (41). First, normalization of the CI was obtained by dividing the CI of a measurement by the CI at a specific time (in this case, 10 min after the addition of the protein). Then, all data were referred to the “baseline CI,” which is the result of subtracting the normalized CI of each well to the normalized CI of the control. As such, the baseline of the control is 0. The cytotoxicity of a protein is described as the decrease below the control in CI after normalization.

### Endpoint confocal laser scanning microscopy (CLSM analysis

HaCaT monolayers subjected to a treatment with different concentrations of 1D10 (20 µM), CecA-linker1-CBD (45 µM), and CecA (45 µM) were analyzed by CLSM after either 40 min or 4 h of incubation. To achieve this, 300 µL of HaCaT cell suspension (10^5^ cells/mL) in DMEM was added to the wells of 8-well µ-Slide chamber (IbiTreat, Ibidi GmbH, Gräfelfing, Germany). Plates were subsequently incubated at 37°C with 5% CO_2_ for 20 h. Next, medium was removed, and the same volume of DMEM with the diluted protein was poured into the well. Experiments were performed in duplicate. As a control, 300 µL of DMEM was added. At each time point (40 min and 4 h), the liquid was removed; cell monolayers were fixed with 4% paraformaldehyde (Sigma Aldrich) in phosphate-buffered saline (PBS) buffer for 10 min, then washed with PBS, permeabilized for 5 min with 0.3% Triton X-100 (Sigma Aldrich) in PBS, and blocked for unspecific binding with 2% bovine serum albumin (BSA) (Fisher BioReagents) in PBS for 30 min. For staining, cells were incubated with anti-ZO-1-AlexaFluor488 (Invitrogen, Thermo Fisher Scientific) and Phalloidin-AlexaFluor568 (Invitrogen) in 0.5% BSA buffer for 1 h at room temperature. After washing, a secondary antibody was used to amplify signal from ZO-1 primary antibody using goat anti-mouse-FITC (Invitrogen), at 1:400 dilution factor in 0.5% BSA buffer, for an additional 1 h at room temperature. DAPI (Sigma Aldrich) was used for nucleic acid staining at 1 µM for 5 min. Finally, images were acquired using a Leica DMi4 confocal microscope using a 63×/NA = 1.4 oil objective and Leica LAS X image software version 3.0.11, using the excitation lasers and emission filters specified for the fluorescent components in the suppliers’ instructions. ImageJ version 1.52o was used for image analysis.

### Statistical analysis

The data obtained from the MIC assays were expressed as the mode of at least three replicates. The time-kill results were expressed as the mean ± SD of three replicates. In all cases, the Student’s *t*-test was used to compare the differences between the treated and untreated bacterial cultures at a level of significance *P* < 0.005 (SPSS-PC+11.0 software, Chicago, IL, USA).

## SUPPLEMENTAL MATERIAL

Supplemental material spectrum.01813-23-s0001.docx Tables S1 to S5, Fig. S1 to S9, and legends for Movies S1 to S7.

Movie S1 spectrum.01813-23-s0002.avi Time-lapse microscopy of killing mechanism induced by 1D10 in dilute buffer.

Movie S2 spectrum.01813-23-s0003.avi Time-lapse microscopy of killing mechanism induced by 1D10 at the poles of the bacterial cell in dilute buffer.

Movie S3 spectrum.01813-23-s0004.avi Time-lapse microscopy of killing mechanism induced by CecA in dilute buffer.

Movie S4 spectrum.01813-23-s0005.avi Time-lapse microscopy of killing mechanism induced by 1D10 in human serum.

Movie S5 spectrum.01813-23-s0006.avi Time-lapse microscopy of killing mechanism induced by CecA in human serum.

Movie S6 spectrum.01813-23-s0007.avi Time-lapse microscopy of killing mechanism induced by CecA-linker1-CBD in dilute buffer.

Movie S7 spectrum.01813-23-s0008.avi Time-lapse microscopy of killing mechanism induced by CecA-linker1-CBD in human serum.

ASM does not own the copyrights to Supplemental Material that may be linked to, or accessed through, an article. The authors have granted ASM a non-exclusive, world-wide license to publish the Supplemental Material files. Please contact the corresponding author directly for reuse.
