# Supplementary material for: Distinct mode of action of a highly stable, engineered phage lysin killing Gram-negative bacteria
Source: Microbiol Spectr. 2023 Nov 16;11(6):e01813-23. doi: 10.1128/spectrum.01813-23 (PMC10714810; doi:10.1128/spectrum.01813-23)
Supplement: Supplemental material — Tables S1 to S5, Fig. S1 to S9, and legends for Movies S1 to S7. [file spectrum.01813-23-s0001.docx]

**Supplementary material**

**Table S1. A) Molecular weight and purification yield of lysin 1D10, CecA-linker1-EAD, CecA-CBD-EAD and CBD-EAD B) SDS-PAGE gels of lysin 1D10, CecA-linker1-EAD, CecA-CBD-EAD and CBD-EAD. Lysin 1D10 and CecA-linker1-CBD were run on a 16% gel, CecA-linker1-EAD, CecA-linker1-CBD, CecA-CBD-EAD and CBD-EAD were run on a 12% gel.**

**A)**

| **Construct** | **Molecular weight (kDa)** | **Yield** |
| --- | --- | --- |
| (Lysin) 1D10  CecA-linker1-CBD-EAD | 29.2 | 22 mg/L |
| CecA-linker1-EAD | 20.1 | 18 mg/L |
| CecA-linker1-CBD | 14.4 | 21 mg/L |
| CecA-CBD-EAD | 28.8 | 6 mg/L |
| CBD-EAD | 24.9 | 24 mg/L |

**B)**

**
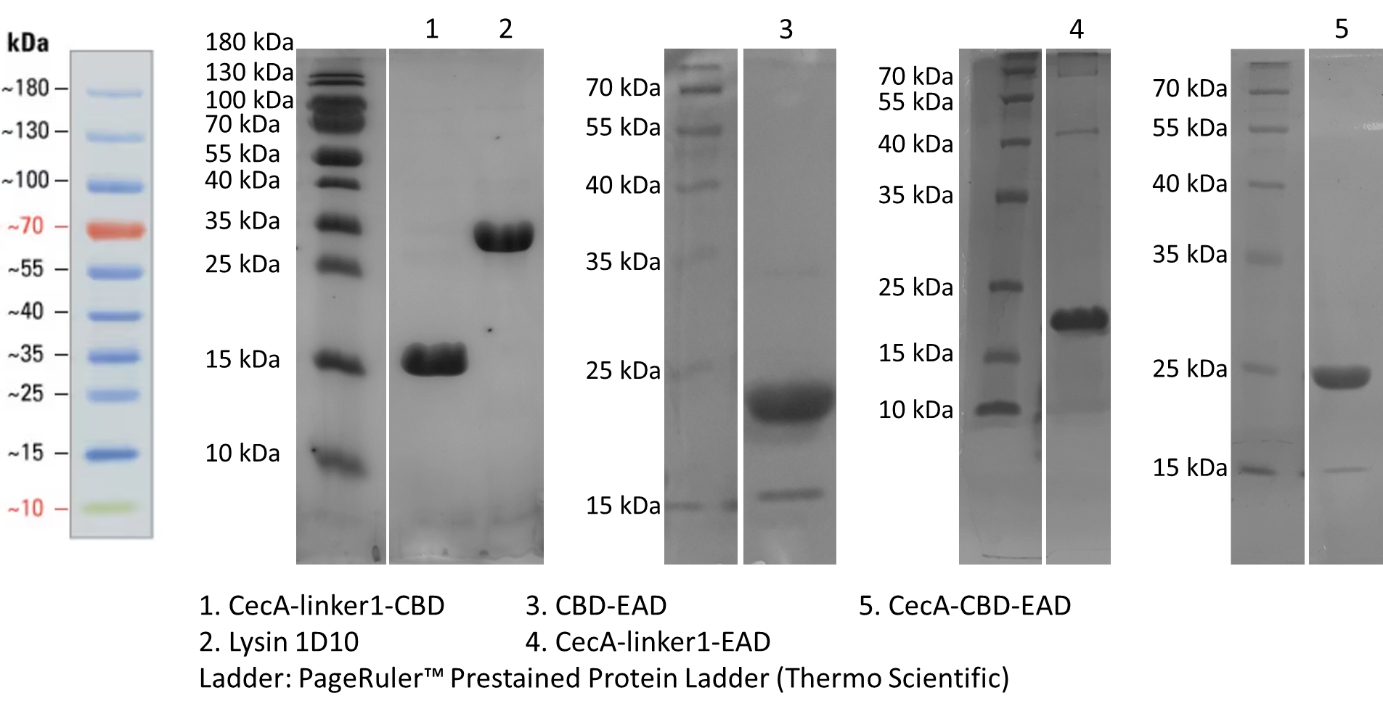
**

**Table S2. A) Main features, molecular weight and purification yield of the 1D10 variants carrying different linkers B) SDS-PAGE gels of the different 1D10 variants. All variants were run on a 12% gel.**

**A)**

| **Name** | **Sequence of linker** | **Properties of linker** | **Linker length (aa)** | **Yield** |
| --- | --- | --- | --- | --- |
| (Lysin) 1D10  CecA-linker1-CBD-EAD | (AG)_3_ | Flexible | 6 | 22 mg/L |
| CecA-linker2-CBD-EAD | (AG)_7_ | Flexible | 14 | 20 mg/L |
| CecA-linker3-CBD-EAD | [16vpA_4](http://zeus.few.vu.nl/programs/linkerdbwww/16vpA_4_b.php), LSRFFHAEL | Rigid, helix | 9 | 5 mg/L |
| CecA-linker4-CBD-EAD | [1clqA_3](http://zeus.few.vu.nl/programs/linkerdbwww/1clqA_3_b.php), VFNQRKEHKGYMLA | Rigid, helix | 14 | 18 mg/L |
| CecA-linker5-CBD-EAD | [1clqA_1](http://zeus.few.vu.nl/programs/linkerdbwww/1clqA_1_b.php), IPQGRSHPVQPYPGAF | Rigid, coil | 18 | 21 mg/L |
| CecA-linker6-CBD-EAD | [1fnf_1](http://zeus.few.vu.nl/programs/linkerdbwww/1fnf_1_b.php), PAVPPP | Rigid, coil | 6 | 16 mg/L |
| CecA-linker7-CBD-EAD | (EAAAK)_3_ | Rigid, helix | 15 | 25 mg/L |

**B)**

**
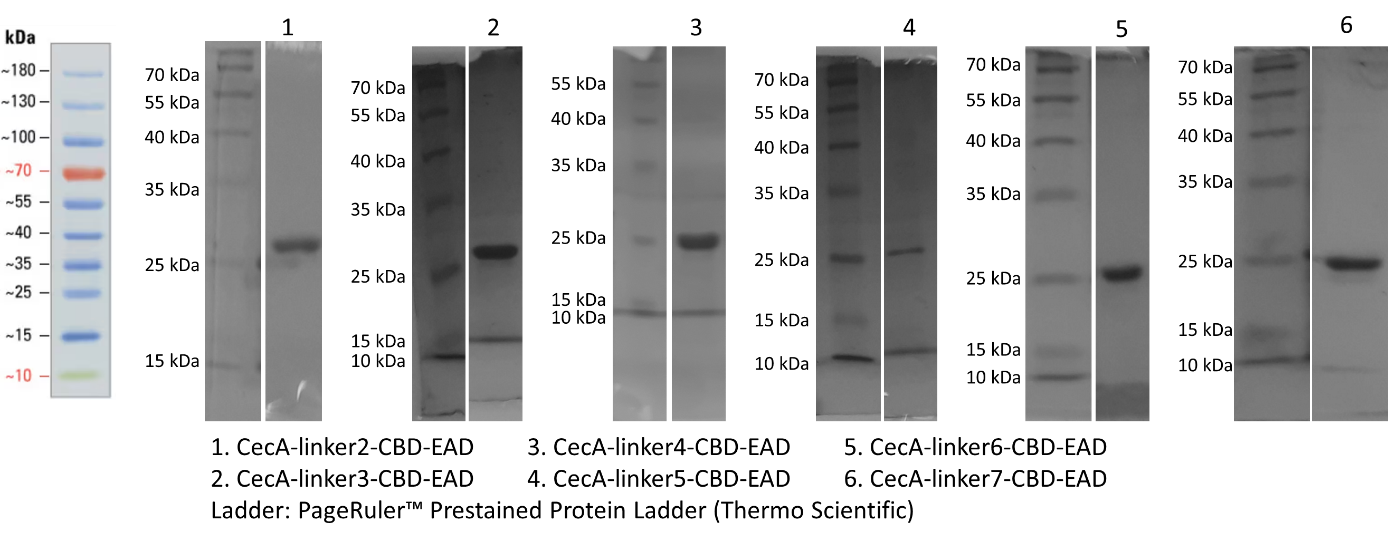
**

**Table S3. MIC values of CecA, CecA-linker-CBD, CecA-linker-EAD and 1D10 in Mueller Hinton medium.** Values represent the mode of three biological replicates. Reference values of MIC/MBCs of *A. baumannii* strains are taken from Table 1.

|  |  |  | **MIC/MBC (µM)** | | | |
| --- | --- | --- | --- | --- | --- | --- |
|  |  |  | **CecA** | | **1D10** | |
|  | **Species** | **Strain** | **0.2 mM EDTA** | **Without EDTA** | **0.2 mM EDTA** | **Without EDTA** |
| Gram - | *A. baumannii* | RUH875 | 0.1 | 0.2 | 0.2 | 0.3 |
| Gram - | *A. baumannii* | RUH134 | 0.1 | 0.2 | 0.1 | 0.4 |
| Gram - | *A. baumannii* | LUH5875 | 0.1 | 0.2 | 0.2 | 0.5 |
| Gram - | *A. baumannii* | NCTC13423 | 0.1 | 0.3 | 0.1 | 0.2 |
| Gram - | *Klebsiella pneumoniae* | ATCC 13883 | 1.1 | 2.2 | > 1.4 | > 1.4 |
| Gram - | *K. oxytoca* | Clinical isolate | 1.1 | > 10.9 | > 1.4 | > 1.4 |
| Gram - | *Pseudomonas* *aeruginosa* | PA14 | 1.1 | > 10.9 | > 1.4 | > 1.4 |
| Gram - | *P. aeruginosa* | PAO1 | 1.1 | > 10.9 | > 1.4 | > 1.4 |
| Gram - | *P. aeruginosa* | Br667 | 1.1 | 8.7 | > 1.4 | > 1.4 |
| Gram - | *E. coli* | ETEC1 O:149 | 1.1 | 3.2 | 1.0 | > 1.4 |
| Gram - | *E. coli* | (APEC)CH2 | 1.1 | 2.2 | 1.0 | > 1.4 |
| Gram - | *S. enteritidis* | ATCC 13076 | 1.1 | 1.1 | 0.5 | > 1.4 |
| Gram - | *S.* Typhimurium | LT2 | 1.1 | 7.6 | 0.1 | > 1.4 |
| Gram - | *S. flexneri* | LMG 10472 | 1.1 | 6.5 | 0.1 | > 1.4 |
| Gram + | *Bacillus subtilis* | sp. subtilis | 1.1 | > 10.9 | > 1.4 | > 1.4 |
| Gram + | *Bacillus cereus* | LMG 9610 | 1.1 | > 10.9 | > 1.4 | > 1.4 |
| Gram + | *Staphylococcus aureus* | VISA/HIP5827 | 3.4 | > 10.9 | > 1.4 | > 1.4 |
| Gram + | *E. faecalis* | HC-1909-5 | > 10.9 | > 10.9 | > 1.4 | > 1.4 |
| Gram + | *Enterococcus sp.* | VRE6 | 1.1 | 4.4 | > 1.4 | > 1.4 |
| Gram + | *E. faecium* | VR-1802-13 | 1.1 | > 10.9 | > 1.4 | > 1.4 |
| Gram + | *S. aureus* | VRS1/HIP11714 | 1.1 | 8.7 | > 1.4 | > 1.4 |
| Gram + | *S. aureus* | ssp.aureus Rosenbach, ATCC 6538 | 2.2 | > 10.9 | > 1.4 | > 1.4 |

**Table S4. Thermoresistance of 1D10 and its derivatives. A.** The effect of different truncations of 1D10 and different linkers on the thermoresistance was evaluated. The proteins were incubated at different temperatures (up to 90 °C) during 30 min and at sterilization conditions (121 °C, 210 kPa for 20 min), followed by a MIC assay in Mueller Hinton against *A. baumannii* NCTC13423. The MIC values are the mode of three biological replicates. The control was carried out at room temperature.

|  | **MIC identical after exposure to** |
| --- | --- |
| **Lysin 1D10** | Sterilization conditions |
| **CecA** | 70 °C |
| **CecA-linker1-CBD** | 50 °C |
| **CecA-linker1-EAD** | Sterilization conditions |
| **CecA-linker2-CBD-EAD** | 80 °C |
| **CecA-linker4-CBD-EAD** | Sterilization conditions |
| **CecA-linker5-CBD-EAD** | Sterilization conditions |
| **CecA-linker6-CBD-EAD** | 70 °C |
| **CecA-linker7-CBD-EAD** | Sterilization conditions |

**Table S5. Primers used in this study.**

| **Purpose** | **Primer sequence (5'→3')** |
| --- | --- |
| Generation of CBD at P1 | Fw: TGTGCTCTTCTAGAGGTCTC**ACCATG**AAAGTATTACGCAAAGGCGATAGG |
|  | Rv: TGTGCTCTTCTCTTGGTCTC**TGCACC**TGCAGTTGGCATAGGGATAG |
| Generation of CBD at P2 | Fw: TGTGCTCTTCTAGAGGTCTC**GGTGCA**AAAGTATTACGCAAAGGCGATAGG |
|  | Rv: TGTGCTCTTCTCTTGGTCTC**ACCTGC**TGCAGTTGGCATAGGGATAG |
| Generation of EAD at P2 | Fw: TGTGCTCTTCTAGAGGTCTC**GGTGCA**CGCCGCGCGCTGCCGTACGAC |
|  | Rv: TGTGCTCTTCTCTTGGTCTC**ACCTGC**GATCCCGCGTGTCGCGAGGTA |
| Generation of EAD at P3 | Fw: TGTGCTCTTCTAGAGGTCTC**GCAGGT**CGCCGCGCGCTGCCGTACGAC |
|  | Rv: TGTGCTCTTCTCTTGGTCTC**GCTTCC**GATCCCGCGTGTCGCGAGGTA |
| Generation of His-tag at P3/P4 | Fw: TGTGCTCTTCTAGAGGTCTC**GCAGGT**CACCATCATCATCATCATTAA |
|  | Rv: TGTGCTCTTCTCTTGGTCTC**TTACTT**TTAATGATGATGATGATGGTG |
| Generation of His-tag at P4 | Fw: TGTGCTCTTCTAGAGGTCTC**GGAAGC**CACCATCATCATCATCATTAA |
|  | Rv: TGTGCTCTTCTCTTGGTCTC**TTACTT**TTAATGATGATGATGATGGTG |
| Generation of linker3 at P2 | Fw: TGTGCTCTTCTAGAGGTCTC**GGTGCA**CTGTCTCGTTTCTTCCACGC |
|  | Rv: TGTGCTCTTCTCTTGGTCTC**ACCTGC**CAGTTCCGCGTGGAAGAAACGA |
| Generation of linker4 at P2 | Fw: TGTGCTCTTCTAGAGGTCTC**GGTGCA**GTTTTCAACCAGCGTAAAGA |
|  | Rv: TGTGCTCTTCTCTTGGTCTC**ACCTGC**CGCCAGCATGTAACCTTTGTG |
| Generation of linker5 at P2 | Fw: TGTGCTCTTCTAGAGGTCTC**GGTGCA**ATCCCGCAGGGTCGTTCTCAC |
|  | Rv: TGTGCTCTTCTCTTGGTCTC**ACCTGC**GAACGCACCCGGGTACGGCTGT |
| Generation of linker6 at P2 | Fw: TGTGCTCTTCTAGAGGTCTC**GGTGCA**CCGGCGGTTCCGCCGCCG |
|  | Rv: TGTGCTCTTCTCTTGGTCTC**ACCTGC**CGGCGGCGGAACCGCCGG |
| Generation of linker7 at P2 | Fw: TGTGCTCTTCTAGAGGTCTC**GGTGCA**GAAGCGGCGGCGAAAGAAG |
|  | Rv: TGTGCTCTTCTCTTGGTCTC**ACCTGC**TTTCGCCGCTTCTTTCGCCGCT |

P: position in VersaTile assembly; Fw: Forward primer, Rv: Reverse primer. BsaI sequences are underlined, while position tags are in bold.

**Figure S1. Amino acid sequence lysin 1D10.** Lysin 1D10 is composed of the Cecropin A peptide produced by the yellow fever mosquito, *Aedes aegypti* (cyan); a neutral, short and flexible linker of three Ala-Gly-repeats (green), the modified CBD of the endolysin of bacteriophage φKZ (purple) and the EAD of gp16 located at the C-terminus of the virion-associated lysin of phage BcepC6B (yellow), followed by a His-tag for purification purposes. The position tags intervening the different building blocks are indicated in bold and underlined. The modifications in the CBD refer to three original cysteine residues that have substituted for serine residues (boxed) to avoid oligomerization. The truncated variants lack one or more of the colored building blocks. In case of the linker variants, the green linker is exchanged by the linkers of Table S2.


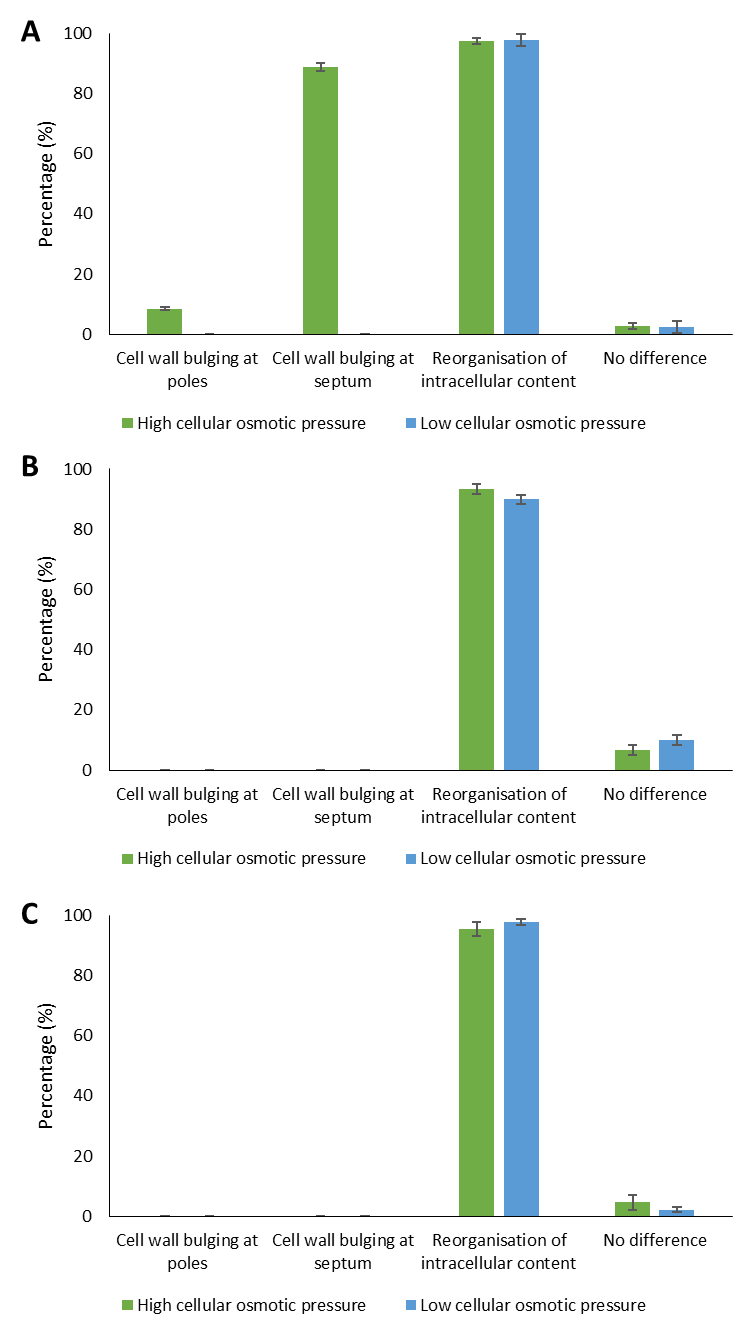


**Figure S2. Distribution of observed events upon exposure to 1D10, CecA or CecA-linker1-CBD.** Microscopic fields covering approximately 100 - 200 *A. baumannii* cells were analyzed for each condition. This was done for three biological replicates. Events were differentiated for cells both under high (green) and low osmotic pressure (blue), undergoing cell wall bulging either at the poles or at the septum, or reorganization of the intracellular content (cell wall bulging was always associated with reorganization of the intracellular content. This is indicated in the graphs as cell wall bulging and reorganization of the intracellular content). If the cells did not show any of these events, they were classified as ‘No difference’. Cells were exposed to equimolar concentrations (2 µM) of **A.** 1D10 **B.** CecA or **C.** CecA-linker1-CBD up to 34 min.


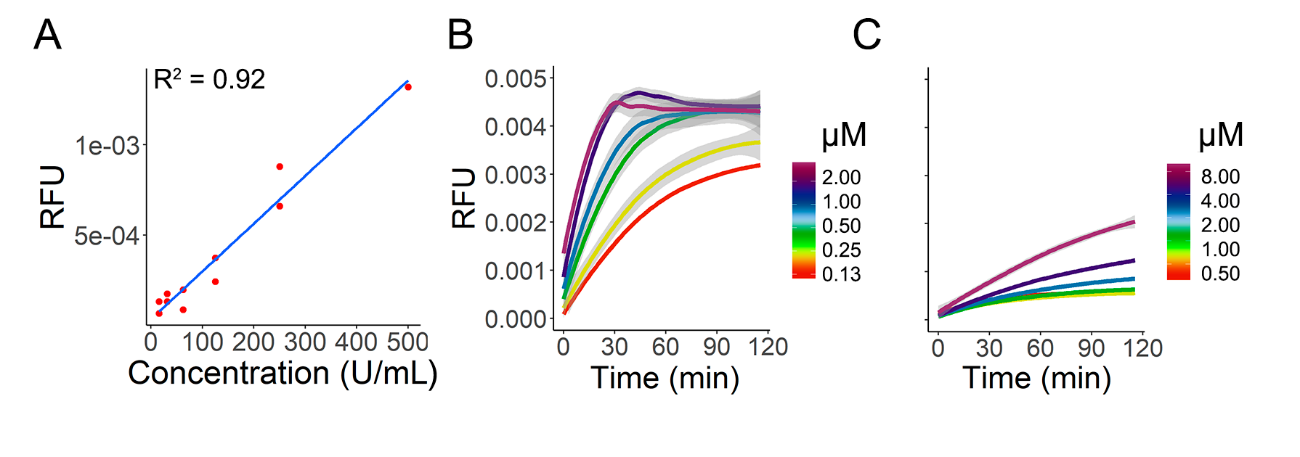


**Figure S3**. Evaluation of the peptidoglycan-degrading activity of 1D10. (A) Calibration curve with a known lysozyme standard. (B) Positive control (hen egg white lysozyme). (C) 1D10. RFU stands for relative fluorescence units. The calibration curve shows the standardized fluorescence measurements at a given time (15 min), and the coefficient of determination for the linear regression of the dataset is provided. In B and C representative results are shown as smoothed kinetics (the grey shade indicates the 95% CI). Further specific activity calculations were performed considering fluorescence measurements of 1D10 and hen egg white lysozyme at the linear region of the kinetics and within the RFU range of the calibration curve.

**Figure S4**. Comparison of turbidity reduction of 1D10 and another engineered lysin eLysMK34 with outer membrane-permeabilized *A. baumannii* RUH134 cells. The control is the same buffer in which 1D10 and eLysMK34 are dissolved (PBS; pH 7.4). The absorbance (OD_600nm_) is measured every 30 s for 1 hour at room temperature.


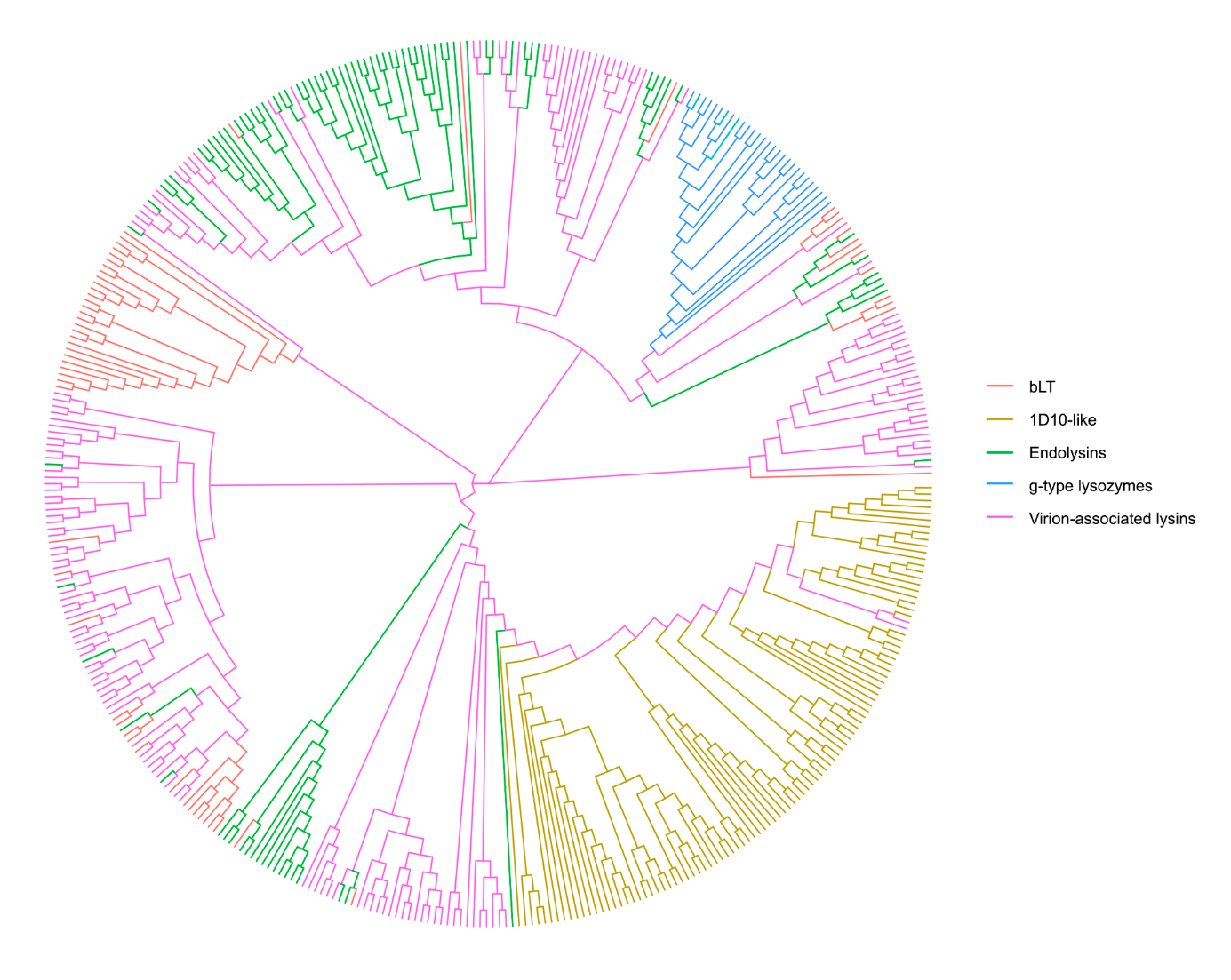


**Figure S5**. Phylogenetic tree of *SLT* family representatives. A sequence dataset was compiled including the representative sequences of the lytic transglycosylase-like sequence cluster at the Conserved Domains Database (cd00254), the g-type lysozyme sequences from the same source (cd01021) and the PhaLP entries (1) with an SLT domain prediction, including both endolysins and virion-associated lysins. The phylogenetic tree was built on a similarity distance matrix obtained from the ClustalOmega-aligned 443 *SLT* sequences using the BIONJ algorithm as implemented in R *ape* library and visualized using *ggtree* library.


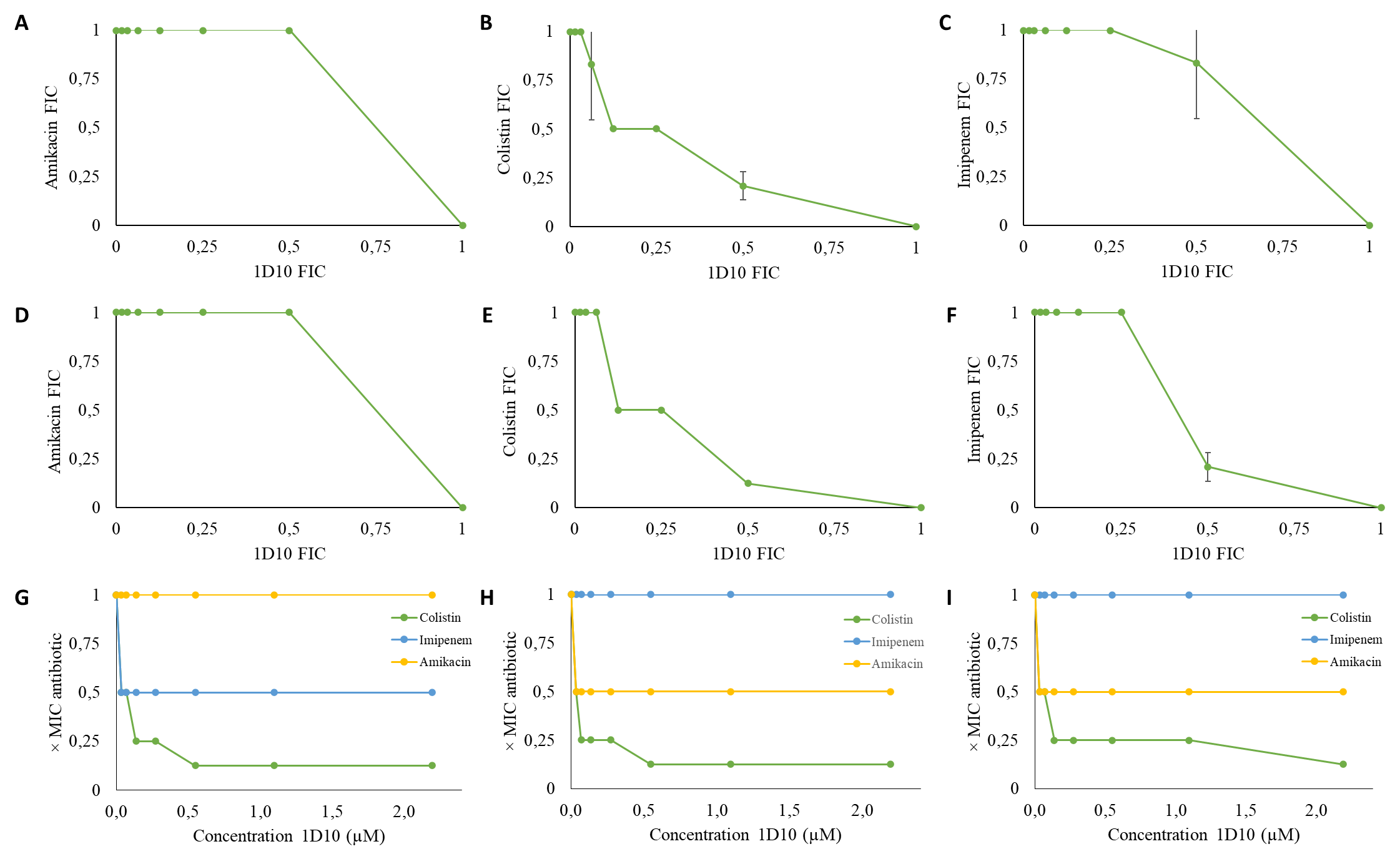


**Figure S6.** Isobologram presenting checkerboard assay of 1D10 in combination with amikacin (yellow), colistin (green) and imipenem (blue) against *A. baumannii* RUH134 (A, B and C, respectively); *A. baumannii* NCTC13423 (D, E and F); *P. aeruginosa* PA14 (G), *P. aeruginosa* Br667 (H), *P. aeruginosa* PAO1 (I). FIC and MIC values represent mode ± standard deviation of three independent replicates.


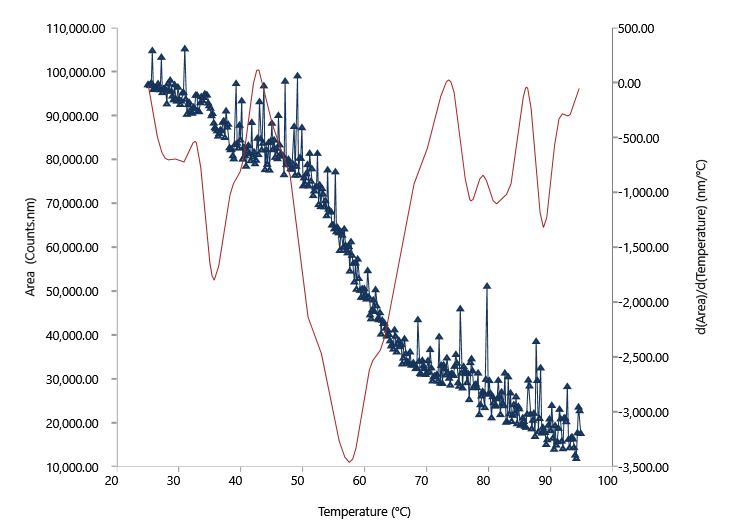


**Figure S7. Unfolding curve of 1D10 and Tm calculation using first derivative.** Thermal unfolding of 1D10 was monitored by recording SYPRO Orange (Thermo Fischer Scientific; Waltham, MA, USA) fluorescence on the UNcle platform (Unchained Labs; Pleasanton, CA, USA) according to the manufacturer’s instructions. The melting temperature (Tm) was calculated from three technical repeats from the first derivative of the area/nm values using the build-in tools of the UNcle platform**.**


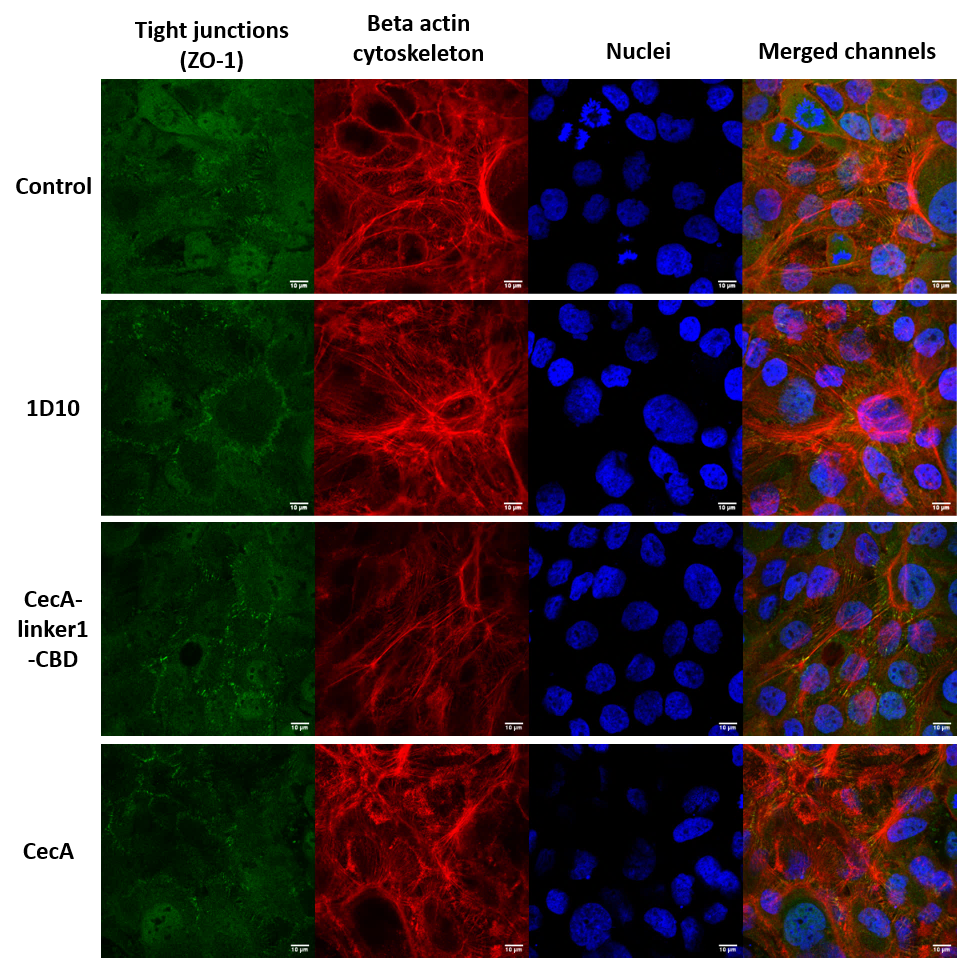


**Figure S8.** Fluorescence images obtained by CLSM of HaCaT after 40 min of incubation with 1D10 (20 µM), CecA-Linker1-CBD (45 µM) and CecA (45 µM); as a control, cells were incubated without protein. The tight junctions, specifically zonula occludes (ZO-1), were labeled with anti-ZO-1-AlexaFluor488 followed by goat anti-mouse-FITC (green). The actin of the cytoskeleton was detected by labeling with Phalloidin-AlexaFluor568 (red) and the nuclei were labeled with DAPI (blue). The scale bars measure 10 µM.

**
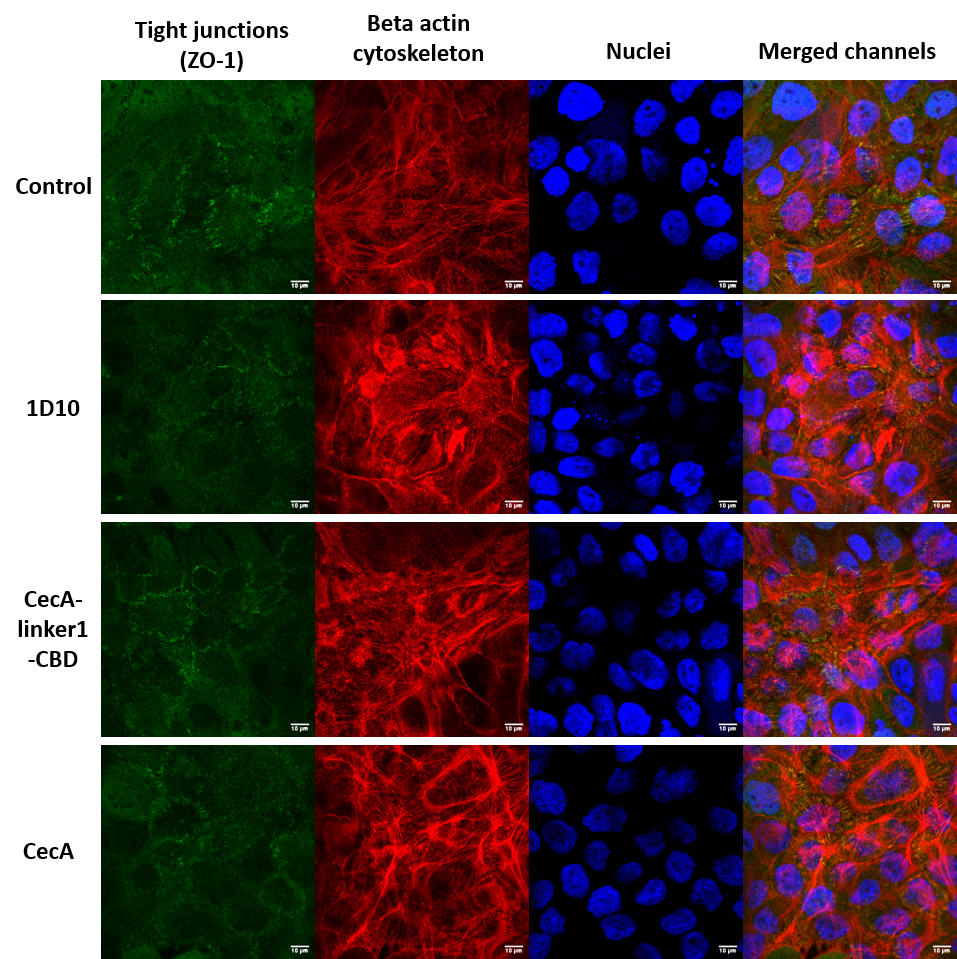
**

**Figure S9.** Fluorescence images obtained by CLSM of HaCaT after 4 h of incubation with 1D10 (20 µM), CecA-linker1-CBD (45 µM) and CecA (45 µM). As a control, cells were incubated without protein. The tight junctions, specifically *zonula occludens* (ZO) 1 were labeled with anti-ZO-1-AlexaFluor488 followed by goat anti-mouse-FITC (green). The actin was detected by labelling with Phalloidin-AlexaFluor568 (red) and the nuclei were labelled with DAPI (blue). The white lines indicate the bar scale of 10 µM.

**Movie S1: Time-lapse microscopy of killing mechanism induced by 1D10 in dilute buffer**. Exponentially growing cells of *A. baumannii* RUH134 with a high osmotic pressure (washed three times with 20 mM HEPES-NaOH pH 7.4) were immobilized on a 1.5 % agar pad made with 20 mM HEPES-NaOH pH 7.4. Frames are taken every 30 s. The bacterial cells display a gradual change in phase contrast over time, starting at the septum. Local cytoplasmic releases are observed at the septum. Scale bar corresponds to 5 µm. The concentration of 1D10 was 2 µM.

**Movie S2: Time-lapse microscopy of killing mechanism induced by 1D10 at the poles of the bacterial cell in dilute buffer**. Exponentially growing cells of *A. baumannii* RUH134 with a high osmotic pressure (washed three times with 20 mM HEPES-NaOH pH 7.4) were immobilized on a 1.5 % agar pad made with 20 mM HEPES-NaOH pH 7.4. Frames are taken every 30 s. The bacterial cells display a gradual change in phase contrast over time, starting at one of either poles. Local cytoplasmic releases are observed at one of the poles. Scale bar corresponds to 5 µm. The concentration of 1D10 was 2 µM.

**Movie S3: Time-lapse microscopy of killing mechanism induced by CecA in dilute buffer**. Exponentially growing cells of *A. baumannii* RUH134 with a high osmotic pressure (washed three times with 20 mM HEPES-NaOH pH 7.4) were immobilized on a 1.5 % agar pad made with 20 mM HEPES-NaOH pH 7.4. Frames are taken every 30 s. The bacterial cells display a gradual change in phase contrast over time, starting at the septum. Scale bar corresponds to 5 µm. The concentration of CecA was 2 µM.

**Movie S4: Time-lapse microscopy of killing mechanism induced by 1D10 in human serum**. Exponentially growing cells of *A. baumannii* RUH134 with a low osmotic pressure (washed three times with 100 % complement inactivated human serum) were immobilized on a 1.5 % agar pad made with 60 % human serum and 40% 20 mM HEPES-NaOH 150 mM NaCl pH 7.4. Frames are taken every 30 s. The bacterial cells display a gradual change in phase contrast over time, starting at the septum. Scale bar corresponds to 5 µm. The concentration of 1D10 was 2 µM.

**Movie S5: Time-lapse microscopy of killing mechanism induced by CecA in human serum**. Exponentially growing cells of *A. baumannii* RUH134 with a low osmotic pressure (washed three times with human serum) were immobilized on a 1.5 % agar pad made with 60 % human serum and 40% 20 mM HEPES-NaOH 150 mM NaCl pH 7.4. Frames are taken every 30 s. The bacterial cells display a gradual change in phase contrast over time, over time, starting at the septum. Scale bar corresponds to 5 µm. The concentration of CecA was 2 µM.

**Movie S6: Time-lapse microscopy of killing mechanism induced by CecA-linker1-CBD in dilute buffer**. Exponentially growing cells of *A. baumannii* RUH134 with a high osmotic pressure (washed three times with 20 mM HEPES-NaOH pH 7.4) were immobilized on a 1.5% agar pad made with 20 mM HEPES-NaOH pH 7.4. Frames are taken every 30 s. The bacterial cells display a gradual change in phase contrast over time. Scale bar corresponds to 5 µm. The concentration CecA-linker-CBD was 2 µM.

**Movie S7: Time-lapse microscopy of killing mechanism induced by CecA-linker1-CBD in human serum**. Exponentially growing cells of *A. baumannii* RUH134 with a low osmotic pressure (washed three times with 100% complement inactivated human serum) were immobilized on a 1.5% agar pad made with 60% human serum and 40% 20 mM HEPES-NaOH 150 mM NaCl pH 7.4. Frames are taken every 30 s. The bacterial cells display a gradual change in phase contrast over time. Scale bar corresponds to 5 µm. The concentration of CecA-linker-CBD was 2 µM.

**References**

1. Criel B, Taelman S, Van Criekinge W, Stock M, Briers Y. 2021. PhaLP: A database for the study of phage lytic proteins and their evolution. Viruses 13:1240.
